# Role and mechanisms of histone methylation in osteogenic/odontogenic differentiation of dental mesenchymal stem cells

**DOI:** 10.1038/s41368-025-00353-z

**Published:** 2025-03-26

**Authors:** Meijun Hu, Zhipeng Fan

**Affiliations:** 1 Beijing Key Laboratory of Tooth Regeneration and Function Reconstruction, Beijing Stomatological Hospital, School of Stomatology, Capital Medical University, Beijing, China; 2 Beijing Laboratory of Oral Health, Capital Medical University, Beijing, China; 3 Research Unit of Tooth Development and Regeneration, Chinese Academy of Medical Sciences, Beijing, China

**Keywords:** Mesenchymal stem cells, Stem-cell differentiation

## Abstract

Dental mesenchymal stem cells (DMSCs) are pivotal for tooth development and periodontal tissue health and play an important role in tissue engineering and regenerative medicine because of their multidirectional differentiation potential and self-renewal ability. The cellular microenvironment regulates the fate of stem cells and can be modified using various optimization techniques. These methods can influence the cellular microenvironment, activate disparate signaling pathways, and induce different biological effects. “Epigenetic regulation” refers to the process of influencing gene expression and regulating cell fate without altering DNA sequences, such as histone methylation. Histone methylation modifications regulate pivotal transcription factors governing DMSCs differentiation into osteo-/odontogenic lineages. The most important sites of histone methylation in tooth organization were found to be H3K4, H3K9, and H3K27. Histone methylation affects gene expression and regulates stem cell differentiation by maintaining a delicate balance between major trimethylation sites, generating distinct chromatin structures associated with specific downstream transcriptional states. Several crucial signaling pathways associated with osteogenic differentiation are susceptible to modulation via histone methylation modifications. A deeper understanding of the regulatory mechanisms governing histone methylation modifications in osteo-/odontogenic differentiation and immune-inflammatory responses of DMSCs will facilitate further investigation of the epigenetic regulation of histone methylation in DMSC-mediated tissue regeneration and inflammation. Here is a concise overview of the pivotal functions of epigenetic histone methylation at H3K4, H3K9, and H3K27 in the regulation of osteo-/odontogenic differentiation and renewal of DMSCs in both non-inflammatory and inflammatory microenvironments. This review summarizes the current research on these processes in the context of tissue regeneration and therapeutic interventions.

Bones and teeth are integral components of the craniofacial skeleton, providing indispensable support for the overlying soft tissues and allowing for complex and intricate movements.^1^ Missing teeth, due to congenital or acquired diseases, and trauma, is by far the most common oral health problem.^2^ Maxillofacial tumors can invade the normal tissue structures of the jaw, teeth, muscles, and skin, severely affecting maxillofacial form and function. The use of autologous or allogeneic bone grafts to repair maxillofacial bone defects caused by tumors or traumatic injuries is common, but problems such as poor osseointegration stability, immune rejection, and interference with normal physiological functions remain. In elderly individuals or those with severe periodontitis, severe damage to the dental tissue can result in malnutrition due to difficulty in eating and chewing, which may subsequently affect an individual’s self-esteem and lead to severe psychosocial and mental health issues.^2^ Prevailing techniques for the restoration of damaged and missing teeth rely on the use of synthetic materials to repair structural defects. However, these techniques do not facilitate the promotion of biological functions such as supplying blood and nerves.

Mesenchymal stem cells (MSCs) are a population of stem cells that function as self-renewing and differentiating progenitor cells. The International Society for Cell & Gene Therapy (ISCT) Mesenchymal Stromal Cell committee defines them as readily adherent, expressing CD105, CD73, and CD90 (but not CD11b, CD14, CD19, CD34, CD45, CD79a, HLA-DR). In addition, these cells are capable of differentiating into osteoblasts, chondroblasts, and adipocyte lineages in vitro.^3,4^ Bone marrow-derived mesenchymal stem cells (BM-MSCs) are a category of seed cells currently employed with considerable frequency in the field of organizational engineering.^5,6^ They are commonly used to repair bone defects owing to their ability to rapidly adapt to the body’s bone microenvironment and accelerate angiogenesis when implanted after mixing with scaffolding materials in vitro. DMSCs have been identified as an optimal cell source for regenerative therapies owing to their excellent proliferative capacity and multidirectional differentiation potential.^7^ Compared to BM-MSCs, DMSCs demonstrate augmented proliferative, neural differentiation, and odontogenic capabilities,^8^ which are highly promising for bone and neural tissue regeneration.^9^ The cellular microenvironment exerts a significant influence on the fate of stem cells and can be modified using various optimization techniques. These include hormone-stem cell combination therapy, gene modification to regulate stem cells, epigenetic regulation, cell membrane sheets, scaffolding materials, and hydrogels.^10^ The transplantation of PDLSCs has been shown to facilitate the regeneration of the cementum, periodontium, and alveolar bone in in vivo experiments conducted on rats, beagles, minipigs, and humans.^9^ In clinical trials, autologous PDLSC cell membrane sheets were used to treat three patients with periodontitis, and it was found that the formation of neoplastic dentin and periodontium was visible around the periodontal tissue.^11^ Dental pulp stem cells (DPSCs) can be combined with collagen sponge scaffolds to form complexes for the repair of human mandibular defects^12^ and the reconstruction of the pulp-dentin complex, thereby generating functional biological tooth roots.^13^ However, current in vitro organoid constructs lack the requisite functional robustness and physiological relevance, particularly in the context of tooth regeneration, which is inherently challenging because of their high structural complexity. Therefore, it is imperative that a potentially effective tissue engineering approach is developed to regenerate teeth that are functional, biocompatible, sustainable, and reproducible, with the ultimate goal of clinical application.^14^

In 1942, Waddington initially proposed that the intricate developmental process from genotype to phenotype could be described by the concept of epigenetics. Epigenetic regulation can alter the chromatin state without modifying the DNA sequence, which, in turn, affects gene expression, protein function, and RNA processing.^15^ Epigenetic modifications can be categorised into three main classifications: These are DNA methylation, histone modifications and chromatin remodelling. DNA methylation is primarily associated with gene regulation and can maintain chromosome integrity. Histone modification is a key process in various biological mechanisms, such as transcriptional regulation, DNA repair, DNA replication, alternative splicing, and chromosome aggregation.^16^

Epigenetic regulation is implicated in a range of biological processes such as embryonic development,^17^ disease onset and progression,^18^ bone homeostasis,^19^ and stem cell fate determination.^20^ A reduction in MSC functionality has been identified as a significant underlying cause of numerous pathological conditions. Epigenetic regulation is of critical importance for the establishment and maintenance of the homeostasis equilibrium of MSCs within the body. Previous studies have demonstrated that osteo-/odontogenic differentiation of DMSCs is regulated by epigenetic modifications.^21,22^ Histone modifications affect osteogenic differentiation by regulating the expression of genes associated with this process. For example, the histone acetyltransferase PCAF has been demonstrated to affect the BMP pathway by elevating histone H3K9 acetylation in the promoter regions of BMP2/4, BMPR1B, and Runx2.^23^ Additionally, histone methylation represents another histone modification that is closely associated with the regulation of osteogenic-related gene expression, either through activation or repression. This paper presents a systematic and comprehensive review of the current research on BM-MSCs and DMSCs in the field of regenerative medicine. We further elucidate the role of epigenetic regulation in tooth regeneration and focus on the role and mechanism of histone methylation in the regulation of osteogenesis-related genes and tooth-derived stem cell functions.

## Osteogenic/odontogenic differentiation-related genes

Bone formation primarily depends on osteoblast differentiation, which is strictly controlled by key transcriptional regulators. These regulators are activated in response to specific external signals and signaling pathways that are regulated during development. The transcription factors (TFs) RUNX2 and SP7 (or Osterix, OSX) are essential for the differentiation of mesenchymal precursor cells into the osteoblast lineage.^24^ Furthermore, the knockout of either RUNX2 or OSX in vivo impedes the normal formation of bone tissue.

### Runx2

RUNX2 is a member of the RUNX family of TFs that forms a heterodimer with core-binding factor b (CBFb). This heterodimer enhances the ability of RUNX2 to bind to DNA and maintains protein stability.^25^ Runx2 is an indispensable regulator of osteoblast and odontoblast differentiation, controlling the expression of specific genes involved in these processes.

Runx2 has the capacity to exert a variety of functions by regulating several key signaling pathways (such as FGF, Hedgehog, Wnt, and Pthlh) and certain TFs (such as OSX and Dlx5).^26^ Wnt signaling has been demonstrated to regulate the function of Runx1 and Runx2.^27^ The canonical Wnt/β-catenin signalling pathway has been shown to regulate osteoblast differentiation. The pathway is initiated by the binding of Wnt to the FZD receptor, which leads to the accumulation of β-catenin. Subsequently, β-catenin moves to the nucleus, leading to the transcription of target genes.^27^ The noncanonical Wnt signaling ligand, WNT5A, has been identified as a key lineage-specific gene in the regulation of differentiation of dental mesenchymal cells. FGFs are a family of secreted peptides controlling a series of secreted peptides in intrachondral and intramembranous ossification.^28^ The Twist, Msx2, and PLZF genes are primarily situated upstream of the Runx2 gene. The principal downstream genes were OSX, ATF4, and ZFP521.^29^ ATF4 has been demonstrated to interact with Runx2, thereby upregulating the expression of osteocalcin (OCN) and Osx.^30^ BMP signaling is essential for Runx2-dependent induction of the osteoblast phenotype.^31^ Upon activation of Smads (Smad1/5/8), BMP2 initiates Runx2 gene transcription through its distal P1 promoter and proximal P1 promoter. Runx2 is also phosphorylated by the non-canonical BMP signaling pathway (TAK1-MEK-p38 or ERK), which promotes its association with the co-activator CREB binding protein.^32^ In addition, BMP also promotes Runx2 acetylation via p300 for Runx2 stabilization.^32^

### Osterix (OSX/Sp7)

OSX/Sp7 is an osteoblast-specific TF expressed in dental embryonic mesenchymal cells. Novel variants of the OSX/Sp7 gene have been identified as a cause of recessive osteogenesis imperfecta, a disorder characterized by bone fragility and hearing impairment.^33^ Sp7/Osx regulates and influences zebrafish larval tooth development and bone mineralization.^34^ During osteoblast differentiation, the molecular switch OSX/Sp7 plays a crucial role in the formation of active chromatin states.^35^ Mice lacking Sp7 are unable to express osteoblast-associated genes such as SPARC(secreted protein, acidic and rich in cysteine), Spp1(secreted phosphoprotein 1)/osteopontin, BSP. Additionally, they exhibited a significant reduction in Cola1 expression, a major component of the bone matrix. Sp7 knockout in mice resulted in serious craniofacial malformations, dentin hypoplasia, and root abnormalities. However, initial tooth morphogenesis and dentin formation in the crowns were not impaired. SP7 is involved in bone and tooth formation through a variety of molecular mechanisms, including FGF and TGF-β/BMP signaling. CHIP-seq data revealed that OSX binds to the BSP promoter, thereby promoting osteoclastic differentiation and mineralization.^36^ SP7 has been demonstrated to promote dentinogenic cell differentiation by upregulating DSPP, DMP1, nestin, and ALP. Furthermore, Sp7 acts as a cofactor of Dlx5 and physically interacts with Dlx5. The Sp7-Dlx5 complex regulates osteoblast differentiation. Other TFs containing homologous structural domains, including Msx1/2, Satb2, and Alx4, may also interact with Sp7 in the osteogenic genome.^34^

### Dentin salivary phosphoprotein (DSPP)

DSPP represents the primary non-collagenous protein present in dentin, osteoblasts, and alveolar bone, and is essential for the normal mineralization of dentin and enamel.^37^ The Runx2 and OSX/Sp7 are essential for the differentiation of osteoblasts and odontoblasts, whereas DSPP exerts a regulatory effect on the latter process.^38^ The functions of DMP1 and DSPP in tooth formation and maintenance can be considered to be complementary and/or synergistic.^39^ It is hypothesized that Runx2 may affect the development of the tooth embryo before the bell stage, yet not during the subsequent stage. Osx may be consistently expressed in adult dentin and pulp cells, stimulating the differentiation and expression of DSPP via a non-Runx2-dependent signaling pathway in the later phase of tooth development. The BMP2 signaling pathway plays a critical role in the differentiation and maturation of odontoblasts. BMP2-deficient teeth display morphological features similar to dentinogenesis imperfecta (DGI), which is associated with mutations in the DMP1 and DSPP genes. Upregulation of BMP2-driven Dlx3, Dmp1, DSPP, and Sp7 promotes mesenchymal cell differentiation and biomineralization.^40^

## The role of histone methylation regulation in the regulation of osteo-/odontogenic differentiation of DMSCs

### Mechanisms of regulation of histone methylation

**Fig. 1:**
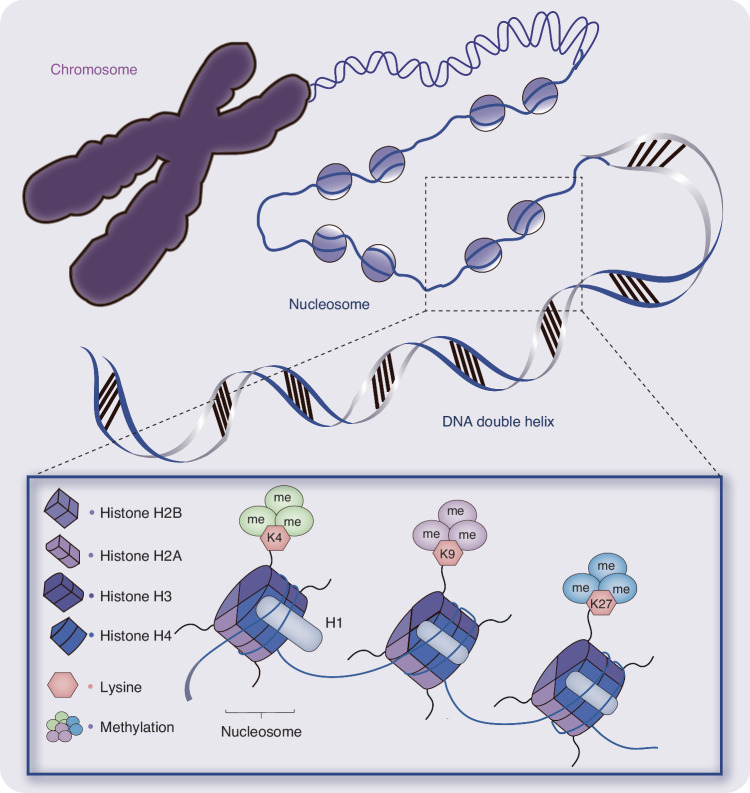
Chromatin and nucleosome structure. Nucleosome is the basic structural unit of chromatin, DNA is wrapped around the nucleosome to form chromatin fibers, and then form chromosomes. Nucleosome consists of a dense octamer composed of histones H1, H2A, H2B, H3, and H4, and connecting region DNA

The basic unit of chromatin is the nucleosome, which is formed by the association of histones (H1, H2A, H2B, H3, and H4) with 147 bp of DNA^41,42^ (Fig. 1). The DNA is densely wrapped in the nucleus of a eukaryotic cell, forming a tightly organized and compact structure that presents a significant challenge to transcription factors attempting to access the genes. The degree of chromatin openness or accessibility is a crucial factor influencing gene expression. This reflects the transcriptional activity of chromatin and represents a pivotal area of investigation in the regulation of gene expression. This field of study holds considerable significance in the context of epigenetic mapping, cell differentiation and development, and the pathogenesis of various diseases.

Histone modifications within promoter regions are indispensable for regulating chromatin accessibility and gene expression.^43^ Amino acid residues located at the N-terminus of histones are susceptible to post-translational modifications, including methylation, acetylation, ribosylation, and ubiquitination, among others.^44^ The pathways involved in these post-translational modifications are clinically approved targets for the treatment of human diseases. Currently, acetylation and methylation have been extensively studied,^45^ and acetylation of histones enhances gene transcription by loosening histone-DNA complexes to allow transcription factor binding,^46^ whereas histone methylation can stimulate or inhibit gene transcription.^47^ Histone methylation has long been implicated in heterochromatin formation and X chromosome inactivation.^48,49^ Dynamic regulation of histone methylation by site-specific histone methyltransferases (HMTs) and demethylases (HDMs) is essential for establishing and maintaining epigenetic modifications.^50^ These enzymes are involved in a variety of essential cellular processes such as cell proliferation and differentiation, DNA damage response and senescence, individual development, and cancer. Histone methylation is primarily observed at arginine (S) and lysine (K) residues within the tails of H3/H4. This is mediated by lysine methyltransferases (PKMTs) and arginine methyltransferases (PRMTs).^50^ Additionally, Lys residues of histones can undergo mono-, dimethyl-, and trimethylation.^51^ The study of lysine methylation sites in mammalian cells has been a prominent area of research, with six sites–K4, K9, K27, K36, and K79 on H3 and K20 on H4–receiving particular attention.^49^ Of these, H3K4, H3K36, and H3K79 have been identified as active markers that occupy the active regions of transcribed genes within chromatin. Conversely, H3K9, H3K27, and H4K20 are regarded as repressive markers that are typically related to silencing of gene expression and condensation of chromatin.^22^ In contrast to H3K9me3, which is a heterochromatin marker, H3K27me3 is usually found in bivalent structural domains together with H3K4me3, and regulates stem cell differentiation. In mammalian cells, H3K27me3 protects heterochromatin tissues following H3K9 methylation deletion.^52^

**Fig. 2:**
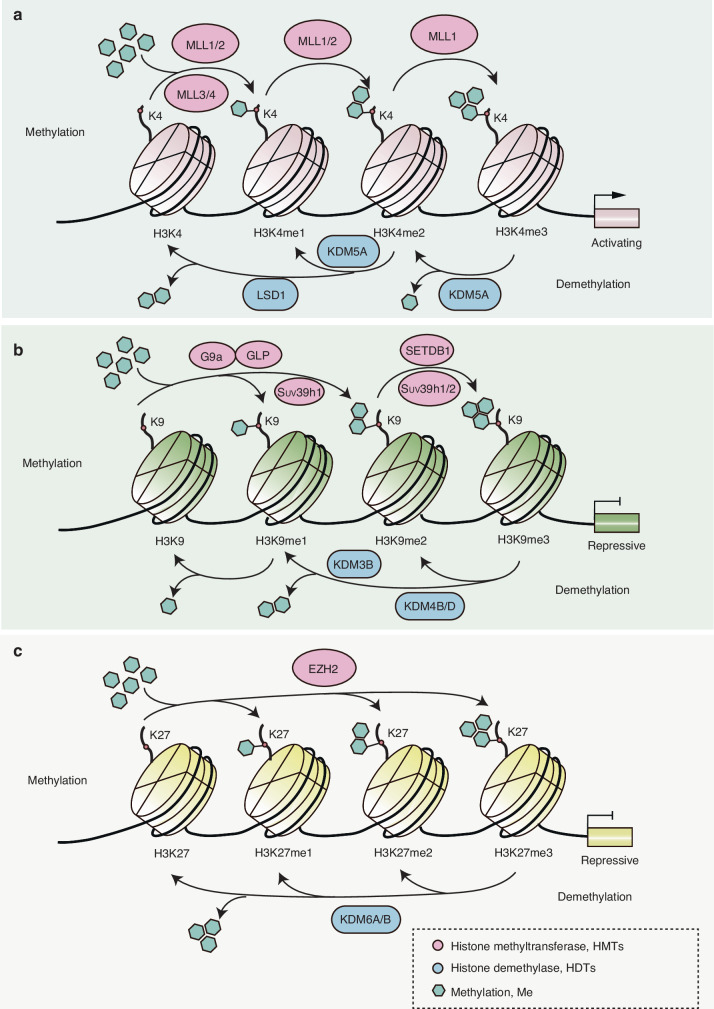
KMTs and KDMs mediate histone methylation modifications. **a** The MLL1/2, MLL3/4 complex catalyzes mono- and dimethylation of H3K4, MLL1 is responsible for H3K4 trimethylation, while KDM5A facilitates the demethylation of H3K4me2 and H3K4me3. LSD1 is responsible for the demethylation of H3K4me1 and H3K4me2. **b** SETDB1 and G9a are involved in the mono/dimethylation of H3K9, while Suv39h1 is responsible for the di-/trimethylation of H3K9. The demethylation of H3K9 is mediated by the JHDM2/KDM3 family, the JHDM3(JMJD2)/KDM4 family, and PHF8/KDM7B; **c** EZH2 is the major lysine methyltransferase of H3K27, while KDM6A/B catalyzes the demethylation of H3K27me1/2/3

**Fig. 3:**
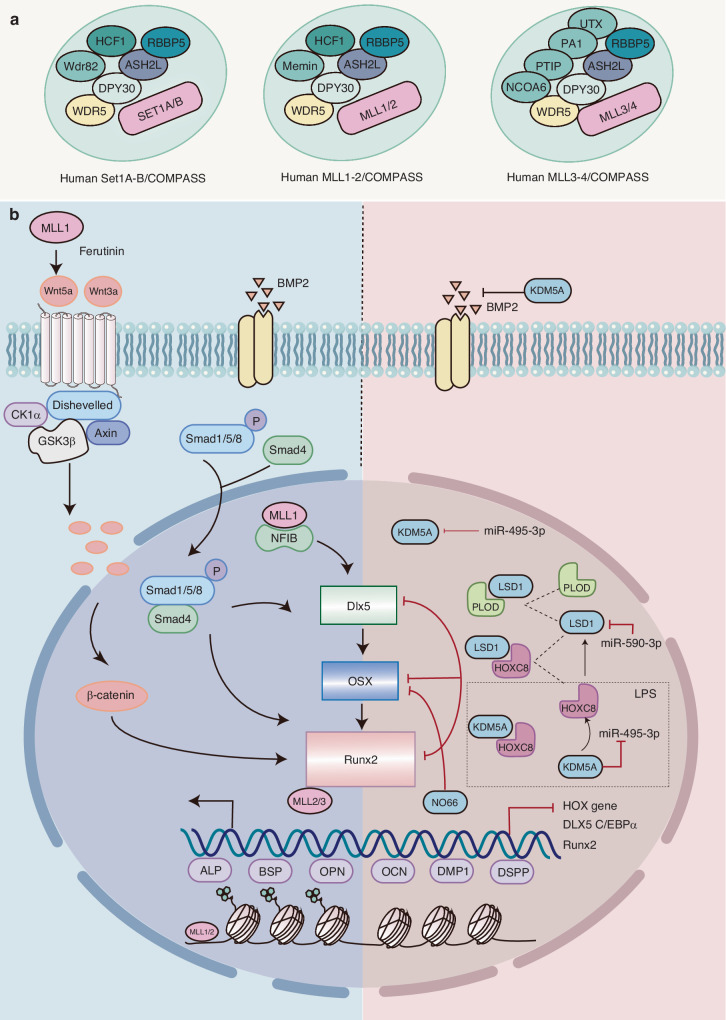
Role of H3K4 histone methylation modifications in osteo-/odontogenic differentiation of bone/dental-derived MSCs. **a** The SET1/COMPASS complex mediated H3K4 methylation, is comprised of MLLs, Set1A/B, WDR5, RbPB5, DYP30, and ASH2. **b** H3K4 histone methylation modifications in osteogenic/odontogenic differentiation MLL1 have been demonstrated to promote Wnt5a transcription and activate downstream transcription factors such as DLX5 and Runx2 by facilitating H3K4me3 generation. This, in turn, affects the transcription of the odontogenic markers DMP1 and DSPP, as well as the genes associated with osteogenic differentiation, OSX, and OCN. Consequently, MLL1 promotes osteogenic differentiation and mineralization of odontogenic stem cells. KDM5A and LSD1 are the major enzymes responsible for demethylating H3K4. Their activity can be influenced by microRNAs, which in turn affects the osteogenic differentiation potential of DMSCs

The most influential histone methylation sites in dental tissues identified in the current study are H3K4, H3K9, and H3K27. For example, dental follicular progenitor cells and alveolar bone osteoblasts display elevated levels of the H3K4me3 mark, whereas periodontal ligament fibroblasts and odontoblasts exhibit high levels of repressive H3K9me3 and H3K27me3 marks on the OSX, IBSP, and BGLAP/Ocn promoters.^47,53^ Specific enzymes are responsible for the methylation of H3K4, H3K9, and H3K27, and their activities are essential for this process. H3K4 methyltransferases can be divided into two main categories: SET structural domain-containing proteins (including Mixed-Spectrum Leukemia Proteins, MLLs, and Set1A/B) and unrelated histone methyltransferases (ASH1, SMYD, and PRDM).^54^ The COMPASS complex, in which mono-, di-, and trimethylates H3K4, comprises MLLs, Set1A/B, WDR5, RbPB5, DYP30, and ASH2. (Fig. 3a) WDR5 is indispensable for the assembly of the SET1 complex and HMT activity. Furthermore, other subunits specifically interact with different KMT2 complexes, thereby increasing the diversity and functional modifications of these methyltransferases. The histone methyltransferases KMT1, KMT2H, and KMT8A/D are involved in methylation at H3K9. The trimethylation of H3K27 is catalyzed by the enzyme EZH2^54^. H3K4 demethylases include the LSD/KDM1 and JARID/KDM5 families. LSD1 removed the methyl group from K4me2 or K4me1, yielding K4me0, whereas KDM5 removed the methyl group from K3me3 or K4me2, producing K4me1.^54^ The histone demethylase classes JMJD1/KDM3, JMJD2/KDM4, and KDM7 are responsible for H3K9 demethylation, whereas KDM6A/B is involved in H3K27 demethylation (Fig. 2).

The impact of histone methylation is determined by the maintenance of a precise equilibrium between H3K4me3, H3K9me3, and H3K27me3, which serve as major trimethylation sites. These modifications are essential for the formation of distinct chromatin structures, which, in turn, regulate the expression of downstream genes.^47,55,56^

### Role of H3K4 histone methylation in osteogenic/odontogenic differentiation

#### MLL family

H3K4 methylation is mediated by the SET1/COMPASS complex, which comprises six catalytic subunits: MLL1-4(KMT2A-D), SETD1A/KMT2F, and SETD1B/KMT2G. The MLL1/2 complex contains the tumor suppressor Menin, which facilitates the recruitment of the MLL1/2 complex to HOX and other sites. The MLL3/4 complex contains NCOA6, PTIP, and PA-1, which contributes to the targeting of the MLL3/4 complex to a distinct set of genes. The MLL3/4 complex also comprises the H3K27 demethylase UTX^57^ (Fig. 3a).

An enhancer is a cis-transcription element critical for the regulation of gene expression. Upon activation, an enhancer binds to various transcription factors, transcriptional co-activators, and RNA Pol II, thereby initiating the activation of target genes. In mammals, H3K4me1 is typically observed in enhancer regions and serves as a hallmark marker of enhancers. Trimethylation of H3K4 (H3K4me3), on the other hand, is predominantly distributed near transcription start sites (TSS) and is associated with transcriptional activation.^58^ H3K4 monomethylation is dependent on the recruitment of MLL3/4. The MLL1/2 core complex is responsible for the conversion of H3K4 to H3K4me1 and H3K4me2. Methyltransferases MLL3(KMT2C) and MLL4 (KMT2D) are indispensable for the activation of enhancers, cellular differentiation, and overall development. They also regulate the early stages of embryonic development and differentiation of embryonic stem cells,^59^ and their functions potentially occur independently of their enzymatic activities.^60^ The MLL2/3-COMPASS complex can bind to the Runx2 P1 promoter, which enriches H3K4me3 levels and decreases H3K4me1 levels. This process has been observed to promote and maintain RUNX2 expression in osteoblasts.^61^ It has been demonstrated that MLL1 is the enzyme responsible for the deposition of H3K4me3 at the promoters of developmental genes. Conversely, the MLL2-COMPASS-like complex has been demonstrated to deposit H3K4me3 at “bivalent” promoters.^60^

Previous research has revealed that the catalytic activity of MLL1 is indispensable to skeletal development.^60^ MLL1 facilitates the deposition of H3K4me3 at the promoter of the Hox gene, a crucial developmental regulator.^62^ Additionally, it has been observed to regulate dental mesenchymal cell differentiation by modulating the formation of H3K4me3 at the WNT5A promoter.^63^ MLL1 forms a functional complex with nuclear factor I family member NFIB and targets the developmental genes DLX5 and Cebpa to promote osteogenic differentiation in MSCs.^64^ SET7 catalyzes H3K4 trimethylation and is significantly expressed in dental papillae at the bell stage, enamel formation stage of developing dental embryos (E17.5, P0 and P3), suggesting that SET7 plays a role in the odontogenic differentiation of dental follicle precursor cells (DFPCs).^65^ The phytohormone, Ferutinin, has been shown to promote the expression of osteocalcin and collagen 1A1 mRNA and protein levels by activating H3K9 acetylation and H3K4 trimethylation markers in the promoter regions of WNT3A and DVL3 and promote the osteogenic differentiation of DPSCs.^66^

Histone demethylation is a crucial epigenetic mechanism in the context of tooth-mandibular regeneration.^67^ The JARID1 family of proteins, LSD1, and the NO66 protein, all of which contain the JmjC structural domain, have been identified as catalysts for H3K4 demethylation. The JARID1 family of proteins demethylates H3K4me2 and H3K4me3, while LSD1 demethylates H3K4me1 and H3K4me2. Additionally, NO66 was identified as a catalyst for all three methylated H3K4 states. These proteins have been observed to exert transcriptional repression through demethylation or recruitment of other inhibitory proteins.

#### KDM5A

JARID1B/KDM5A has been demonstrated to remove H3K4me2 and H3K4me3, thereby repressing transcription of the Runx2 P1 promoter of osteogenesis-related genes. This results in the inhibition of osteogenic differentiation and exerts a negative regulatory role in bone formation in osteoporotic mice.^68,69^ KDM5A binds to the promoters of RUNX2, OCN, and OPN, resulting in reduced levels and downregulation of H3K4me3. Several miRNAs have been shown to regulate the function of KDM5A, which in turn regulates osteogenic differentiation. Examples of such miRNAs include miR-107^69^ and miR-29b-3p.^70^ During the odontogenic differentiation of human dental pulp cells (hDPCs), there was a notable increase in mRNA expression of KDM5A at the initial stage and in a time-dependent manner throughout the induction of tooth formation. Knockdown of KDM5A resulted in elevated expression of DMP1, DSPP, OSX, and OCN. A notable increase in total H3K4me3 levels was observed, whereas H3K4me2 levels remained unaltered. It has been hypothesized that KDM5A may inhibit the odontogenic differentiation of hDPCs by removing H3K4me3 from the promoters of specific genes. This suggests that KDM5A-dependent histone demethylation is a crucial factor in restorative dentinogenesis.^71^

#### LSD1

Lysine-specific demethylase 1 (LSD1, also designated as KDM1A) is a member of the flavin adenine dinucleotide (FAD)-dependent amine oxidase family of demethylases. LSD1 is responsible for removing mono- and bi-methylation modifications of histones H3K4 and H3K9.^72^ As an initial member of the histone demethylation family, it plays a crucial role in the differentiation of stem cells. LSD1 occupies the binding site for H3K4me2/3, and its activity is essential for the normal formation of bone tissue.^73^ The administration of LSD1 inhibitors have been demonstrated to prevent osteoporosis after ovarietomy (OVX) in mice by increasing the number of osteoblasts.^74^ KDM1A/LSD1 has been shown to exhibit dynamic bidirectional effects during osteogenic/odontogenic differentiation of DMSCs. Knockdown of KDM1A increased the expression of BSP, DSPP, DMP1, OSX, Runx2, and DLX in SCAP. Conversely, in an in vivo transplantation experiment, more mineralized tissue formation was observed in the KDM1Ash group than in the control group, accompanied by upregulated expression of BSP and DSPP. Further studies demonstrated that KDM1A can form a protein complex with PLOD2, an important gene involved in the osteogenic differentiation of BM-MSCs, and that this complex can inhibit the osteo-/odontogenic differentiation of SCAP.^75^ HOXC8 gene has been found to controls the differentiation of spinal motor neurons.^76^ HOXC8 has been seen to binds directly to the KDM1A promoter region, thereby regulating KDM1A. This results in a negative regulation of the osteo-/odontogenic differentiation of SCAP and the expression of Runx2 and Osx.^77^

The JmjC structural domain-containing NO66 is a histone demethylase that can catalyze the removal of mono-, di-, and trimethyl marks at H3K4, di- and trimethyl marks at H3K36. In mammals, NO66 is important for bone formation during early development and maintenance of adult bone homeostasis. During osteogenesis induction, NO66 has been observed to interacts with OSX, thereby inhibiting its transcriptional activity and suppressing osteogenesis. This is achieved by removing labeling from the promoters H3K4me3 and H3K36me3 of OSX target genes, including bone salivary protein (BSP), which in turn inhibits osteogenesis.^35,78^ Although NO66 has been shown to inhibit osteoblast differentiation, whether it plays a similar role in the differentiation of DMSCs into odontoblasts remains unanswered.

#### Inflammatory condition

During the process of inflammation, various inflammatory factors (e.g., TNF-a, IL-1, IL-6) have been observed to affect the epigenetic characteristics of cells, thereby influencing gene expression.^79^ Moreover, the inflammatory microenvironment has been demonstrated to impact stem cell differentiation and function.

In patients with chronic periodontitis, bacteria can alter histone modifications in periodontal tissue and activate a series of inflammatory and bone metabolism-related signaling pathways.^80,81^ Lipopolysaccharide (LPS) has been identified as a pivotal periodontal pathogenic factor, capable of activating NF-κB signaling and promoting the release of inflammatory cytokines such as IL-6 and IL-8. Research has demonstrated that rat dental capsule stem cells possess the capacity to modulate the expression of ERK1/2 and NF-κB signaling pathway via the paracrine pathway. This modulation involves the repression of IL-1β, IL-6, and TNF-α gene expression, while concurrently promoting the expression of IL-4 and TGF-β. This regulatory mechanism contributes to the preservation of dental pulp regeneration in rats afflicted with inflammation.^82^ In response to LPS stimulation, there was an increase in H3K27me3 enrichment in the extracellular matrix and osteogenic gene promoters, whereas H3K4me3 enrichment increased in the inflammatory response gene promoters.^83^ In an LPS-induced periodontitis model, the histone lysine methyltransferase SETD1 promoted the expression of inflammatory genes (such as IL-1β, IL-6, and MMP2) in PDLSCs through H3K4 trimethylation.^80^ SETD1 and p65 have been shown to have a synergistic role in inflammatory regulation. Additionally, LPS increased the intranuclear localization of SETD1, which, in turn, mediated the nuclear translocation of p65 through SETD1 in a p65-dependent manner. Furthermore, the use of an NF-κB inhibitor has been shown to reduce SETD1B expression and enhance osteogenesis in vivo. The expression of KDM5A and LSD1 was increased in LPS-treated hPDLSCs.^84^ KDM5A knockdown enhanced ALP activity, promoted mineralization, and upregulated the expression of Runx2, OCN, and OPN. ChIP analysis confirmed that KDM5A binds to the miR-495-3p promoter and inhibits the expression of miR-495-3p by demethylating H3K4me3, thereby enhancing HOXC8 transcription and inhibiting the osteogenic differentiation, proliferation, and migration of hPDLSCs in patients with periodontitis.^85^ miR-590-3p targets LSD1 transcription, upregulates H3K4me2 methylation, and promotes OSX transcription, which in turn facilitates the osteogenic differentiation of hPDLSCs in periodontitis.^84^

Markers of H3K4me3 activity are present in the promoters of TFs of the early osteogenic lineage, including RUNX2, MSX2, and DLX5. The regulation of histone demethylases/methylases may affect the level of H3K4me2/3 at the promoters of osteo-/odontogenic genes, which in turn affect the transcription of DMP1, DSPP, OSX, and OCN, thereby affecting the osteo-/odontogenic differentiation of DMSCs. KDM5A and LSD1 are the major demethylases of H3K4 (Table 1). Modification of these enzymes or the utilization of miRNAs to affect their transcription may represent promising targets for enhancing dental-derived stem cell-mediated tissue regeneration (Fig. 3b).

**Table 1:** Role of H3K4 histone methylation modifications in Osteo-/Odontogenic differentiation of bone/dental-derived MSCs

Epigenetic modification factors		Epigenetic marker	Cell	Function	Reference
Normal	MLL1	H3K4-H3K4me2/3	hDPCs	MLL1 facilitates the formation of H3K4me3 and enhances the transcription of Wnt5a	^63^
	SETD7	H3K4-H3K4me3	DFPCs	SETD7 exhibits significantly upregulated expression in dental papillae at E17.5, P0, and P3 stages, playing a crucial role in the odontogenic differentiation of DFPCs	^65^
	-	H3K4me3	DPSCs	Ferutinin activates typical Wnt/β-catenin signaling pathway and promotes osteogenic differentiation of DPSCs	^66^
	KDM1A/LSD1	H3K4me2/1↓, H3K9me2/1↓	hSCAP	KDM1A interacts with PLOD2 to form a protein complex, thereby playing a dynamic role in the regulation of osteogenic differentiation of hSCAP	^75^
		H3K4me2↓	hSCAP	The expression of KDM1A was enhanced by HOXC8, while the expression of RUNX2 and OSX was inhibited. Furthermore, odontogenic differentiation, migration, and chemotactic ability were all inhibited in hSCAP.	^77^
	LSD1 inhibitor, pargyline	H3K4me2	BM-MSCs	Increasing H3K4me2 levels in the Runx2 promoter region promotes osteogenic differentiation	^74^
	KDM5A	H3K4me3↓	hDPCs	Removal of H3K4me3 and inhibition of the expression of osteogenic differentiation genes DSPP, DMP1, OCN, and OSX	^71^
	KDM5A	H3K4me3↓	BM-MSCs	KDM5A removed H3K4me3 labeling from RUNX2 and inhibited the expression of BMP2 and osteogenic differentiation	^68^
Inflammatory conditions	SETD1	H3K4- H3K4me3	PDLSCs	SETD1B plays a role in p65 nuclear translocation, the upregulation of expression of genes involved in inflammation (IL-1β, IL-6, and MMP2), and the inhibition of osteogenesis.	^80^
	KDM5A	H3K4me3↓	hPDLSCs	KDM5A binds to the miR-495-3p promoter and inhibits its expression, enhances HOXC8 transcription, and inhibits osteogenic differentiation, proliferation, and migration of hPDLSCs	^85^
	LSD1	H3K4me2↓	hPDLSCs	LSD1 suppresses OSX transcription by demethylating H3K4me2, while miR-590-3p regulates LSD1 expression to enhance osteogenic differentiation of inflammatory hPDLSCs	^84^

### Role of H3K9 histone methylation in osteogenic/odontogenic differentiation

Notably, more than 50% of vertebrate genomes are packaged as condensed and transcriptionally repressed heterochromatin.^86^ Epigenetic markers of heterochromatin are H3K9me2 or H3K9me3. H3K9 methylation is a key epigenetic mark, and this modification has been associated with tissue-specific gene silencing. Theoretically, H3K9 methylation and chromatin densification inhibit transcription in three ways. First, they restrict the entry of RNA polymerase complexes. Second, they can limit the elongation of the transcript or stability of the mRNA. Third, they can block pathways or functions that activate transcription factors.

#### G9a, GLP, PRDM2, and SUV39H1

**Fig. 4:**
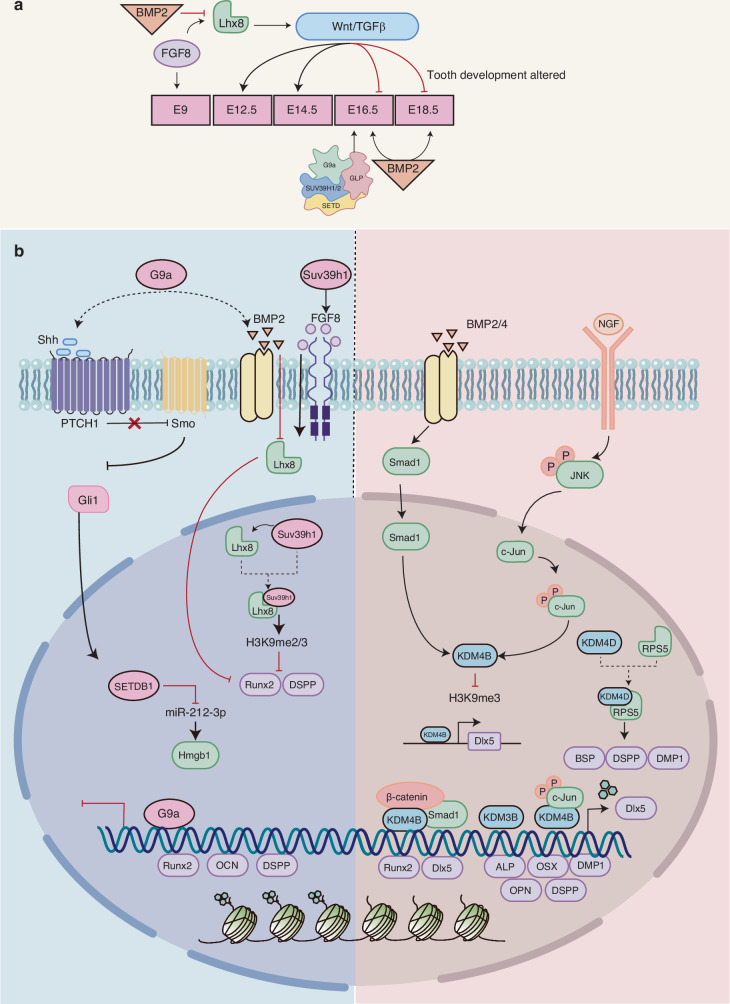
Role of H3K9 histone methylation modifications in osteo-/odontogenic differentiation of bone/dental-derived MSCs. **a** The synergistic expression of G9a, GLP, PRDM2, and SUV39H1 regulates the expression of signaling molecules involved in mesenchymal-epithelial interactions and tooth development. The deletion of G9a may result in a delayed expression of Shh, and BMP2/4 in the tooth embryo. The Lhx8/Suv39h1 complexes are reciprocally regulated by epithelial-mesenchymal signaling, thereby maintaining a balance between interstitial differentiation and proliferation through H3K9 methylation. **b** Setdb1 and G9a have been demonstrated to inhibit osteogenic differentiation of DMSCs. However, KDM4B/D and KDM3B have been shown to promote osteo-/odontogenic differentiation of DMSCs by regulating the expression of specific genes

In mammals, H3K9 methyltransferases with different catalytic activities and target genes are involved in various cellular processes. These enzymes include the SUV39H1/2, dimeric G9a-GLP, and PRDM families. Synergistic expression of G9a, GLP, PRDM2, and SUV39H1 during mouse dental embryo development is critical for the regulation of tooth development. The differential distribution of these factors in the mesenchyme may be associated with the epigenetic regulation of signaling molecules engaged in mesenchymal-epithelial interactions. SETDB1 catalyzes the monomethylation of H3K9 in the periplasmic region. Furthermore, evidence suggests that SETDB1 may inhibit osteogenic differentiation of BM-MSCs, regulate osteogenic differentiation through the Setdb1/miR-212-3p/Hmgb1 pathway in hFOB cells.^87^ G9a may serve as a catalyst for mono/dimethylation of H3K9.^88^ G9a deficiency may result in delayed expression of Shh and BMP2/4 in dental embryos. In addition, G9a may bind to Runx2 to form a complex, which is recruited to the endogenous Runx2 binding site and is directly involved in regulating tooth development.^89^ In pulp-derived MSCs, low levels of G9a reduce inhibitory H3K9me2 production and promote osteogenesis.^90^ SUV39H1/2 catalyzes the dimethylation and trimethylation of H3K9 in constitutive heterochromatin. LIM homobox8 (Lhx8) is a highly conserved transcription factor that regulates dentin development and regeneration through the Wnt and TGF-β pathways.^91^ Elevated Lhx8 levels facilitate the activation of these two pathways, thereby ensuring the maintenance of mesenchymal development during the initial stages of tooth development. Conversely, the differentiation of odontoblast cells is inhibited by Wnt and TGF-β in the subsequent phase. Through RNA hybridization, Zhou et al. discovered that the co-expression pattern of Lhx8 and Suv39h1 in the mesenchyme was consistent with the dynamic expression profiles of the early epithelial signaling molecule FGF8 and the later mesenchymal signaling molecule BMP2.^92^ In hDPSCs, Suv39h1 forms a complex with Lhx8, which inhibits the expression of the tooth-forming genes Runx2 and DSPP by controlling the formation of H3K9me2/3. (Fig. 4a) The PRDM family members are also involved in H3K9 methylation. Although Prdm3 and Prdm16 have been demonstrated to have a significant impact on craniofacial development by maintaining the spatiotemporal expression of genes associated with cranial neural crest cell development,^93^ their involvement in DMSCs differentiation has seldom been investigated and the underlying mechanisms remain unclear.

#### JHDM3(JMJD2)/KDM4

The primary enzymes implicated in the demethylation of H3K9 are members of the JHDM2/KDM3, JHDM3(JMJD2)/KDM4, and PHF8/KDM7B families.^94^ The JHDM2A-C proteins mediate the demethylation of H3K9me1/2, thereby regulating hormone-dependent transcriptional activation. In addition, JHDM3 catalyzes the demethylation of H3K9me2 and H3K9me3 in vitro. KDM3A and KDM4C have been shown to regulate heterodimerization through transcriptional activation of the lectin components NCAPD2 and NCAPG2. Chromatin reorganization inhibits progression of DNA damage and cell senescence.^95^ KDM4B was identified as an epigenetic coordinator of β-catenin/Smad1-mediated transcription, facilitating the removal of the repressive marker H3K9me3.^96^ JMJD2/ KDM4B regulates the expression of H3K9me3 in the Runx2 promoter region. Its deletion inhibits osteogenic differentiation of MSCs isolated from oral bones (OMSCs) and promotes oral and maxillofacial bone senescence.^97^ JMJD2B/ KDM4B facilitates the osteogenic differentiation of hBM-MSCs via regulating the methylation level of H3K9me2 on the Runx2 promoter.^98^ DLX homologous proteins are involved in the formation of functional dental epithelial tissues and crown morphogenesis, regulating the differentiation of odontoblast cells.^99^ KDM4B binds to the DLX5 promoter and removes H3K9me3 from the DLX5 gene promoter, and DLX5 plays a key role in osteogenic differentiation by regulating OSX expression.^100^ The DLX5 gene is highly expressed in DMSCs. DLX5 and KDM4B exert positive effects on BMP signaling. These two factors appear to regulate each other through a positive feedback mechanism.^101^ The induction of Osterix expression by BMP-2 is facilitated by Dlx5, yet it functions independently of Runx2. This mechanism promotes the osteo-/odontogenic differentiation potential of SCAP by promoting the expression of ALP, DSPP, DMP1, OPN, and OSX.^102^ In addition, KDM4B can act in conjunction with c-Jun within the JNK signaling pathway, facilitating its recruitment to the DLX5 promoter region, thereby regulating the NGF-mediated osteogenic differentiation of DMSCs.^101^ KDM4D enhanced the migratory and chemotactic capabilities of SCAP. Using protein profiling technology, co-binding proteins of KDM4D and ribosomal protein S5 (RPS5) were successfully identified. The KDM4D-RPS5 complex jointly promotes osteo-/odontogenic differentiation potential and migratory capacity of SCAP by increasing the expression of ALP, DSPP, and DMP1. These findings suggest that KDM4D is a promising candidate for dentin tissue.^103^ KDM3B/JMJD1B is a demethylase that alters chromatin modifications through demethylation of H3K9me2. This demethylation process promotes the osteo-/odontogenic differentiation of SCAP by upregulating the expression of specific genes (such as Runx2, Osx, Ocn, and Dspp)^67^ (Fig. 4b).

#### Inflammatory condition

While the epigenetic mechanism of KDMs plays a pivotal role in numerous biological processes, evidence suggests that KDM3C exerts an anti-inflammatory effect on oral bacterial infections in periapical and periodontal tissues through the inhibition of NF-κB signaling and osteoclast (OC) production.^104^ However, its role in the inflammatory response to oral bacterial infections remains unclear.

H3K9me3 is predominantly associated with transcriptional silencing and occupies the promoter region. KMTs and KDMs determine histone methylation and are associated with osteogenic differentiation. KMTs are involved in the repression of the histone marker H3K9, which includes Setdb1 and G9a, and have been demonstrated to inhibit the osteogenic differentiation of DMSCs. However, KDM4B/D and KDM3B have been shown to promote the osteo-/odontogenic differentiation of DMSCs by regulating the expression of specific genes (Table 2).

**Table 2:** Role of H3K9 histone methylation modifications in Osteo-/Odontogenic differentiation of bone/dental-derived MSCs

Epigenetic modification factors		Epigenetic marker	Cell	Function	Reference
Normal	G9a	H3K9- H3K9me2	hDP-MSCs	G9a facilitates the generation of H3K9me2 and suppresses the transcription of osteogenic markers, including Runx2, Ocn, and DSP.	^80^
	Suv39h1	H3K9- H3K9me2/3	DPSCs	Suv39h1 forms a protein complex with Lhx8, which functions to inhibit the expression of odontogenic genes through the di/trimethylation of H3K9.	^92^
	KDM4B	H3K9me3	hSCAP	DLX5 interacts with KDM4B and facilitates osteo-/odontogenic differentiation through the upregulation of OSX.	^102^
		H3K9me3	DMSC	The P75^NRT^-mediated NGF signaling pathway induces the activation of the JNK cascade and KDM4B expression through the removal of inhibitory H3K9me3 marks, thereby stimulating DLX5 and subsequently promoting osteogenic differentiation.	^101^
	KDM4D	H3K9me2/3	hSCAP	The combination of KDM4D and RPS5 promotes osteo-/odontogenic differentiation and the migration capacity of SCAP	^103^
	KDM3B	H3K9me2	hSCAP	KDM3B upregulates the expression of Runx2, Osx, Ocn, and Dspp genes, thereby promoting osteo-/odontogenic differentiation in SCAP.	^67^

### Role of H3K27 Histone methylation in Osteo-/Odontogenic differentiation

H3K27me3 is associated with gene silencing and predominantly deposited in CpG-rich promoters. H3K27me3-labeled promoters can still be bound by general transcription factors and by suspended Pol II binding, and are therefore considered parthenogenetic heterochromatin, which exhibits a mechanistic profile distinct from that of H3K9me3.

**Fig. 5:**
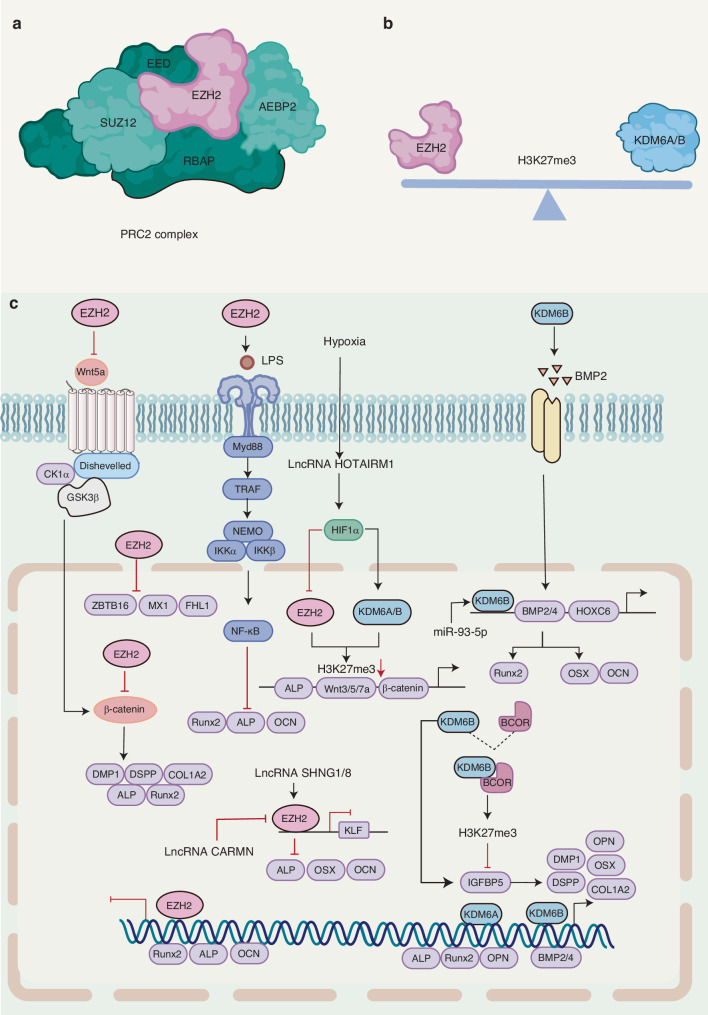
Role of H3K27 histone methylation modification in osteo-/odontogenic differentiation of bone/dental-derived MSCs. **a** PRC2 protein complex. **b** EZH2 has been demonstrated to antagonize the function of KDM6A/B and to co-regulate the level of H3K27me3. **c** PRC2 core subunit EZH2 has been shown to catalyze H3K27 trimethylation and maintain gene silencing, thereby inhibiting the osteogenic differentiation of DMSCs. The specific demethylase KDM6A has been demonstrated to enhance the osteogenic differentiation potential of DMSCs by removing the H3K27me3 mark at the Runx2 and Ocn transcription start sites. KDM6B is required for osteogenic differentiation, with KDM6B binding to BMP2 and HOX genes and co-regulating downstream OSX, BGLAP, and SPP1 transcription

H3K27 methylation is mediated by the Polycomb Repressive Complex 2 (PRC2), which is composed of four core subunits: EZH2, SUZ12, EED, and RBAP46/48.^49,105,106^ (Fig. 5a) EZH2, the core component of PRC2, facilitates the addition of a methyl group to histone H3K27, ultimately contributing to the maintenance of gene silencing.^107^ SUZ12 and EED are essential for the enzymatic activity of EZH2. EZH2 contains a carboxy-terminal catalytic SET structural domain. During embryonic development, EZH2 reduces chromatin accessibility and promotes silencing of HOXA and HOXD cluster genes through H3K27 trimethylation-catalyzed nucleosome compaction.^108,109^

#### EZH2

Numerous studies have identified that Ezh2 as a key regulator of osteogenic differentiation and skeletal development.^110,111^ Overexpression of EZH2 facilitates lipogenic differentiation of MSCs.^112^ Under osteogenic induction conditions, EZH2 expression is downregulated and segregated from the RUNX2 promoter. Chromatin accessibility in osteoblasts may be maintained through the synergistic action of Runx2 and its potential co-regulators, including Sp7 and AP-1, on enhancer modules that regulate osteoblast development.^113^ The conditional knockdown of Ezh2 in undifferentiated MSCs resulted in defects in skeletal morphology and bone formation.^114,115^ EZH2 exerts its effects not only by directly regulating signaling modules and specific TFs, but also through targeting of numerous novel genes(including ZBTB16, MX1, and FHL1) that mediate the osteogenic differentiation of MSCs^110^ As a histone methylation transferase, EZH2 enhances migration and chemotaxis of DMSCs.^116^ Conversely, evidence indicates that it negatively regulates odontogenic differentiation.^117–119^ EZH2 has been observed to inhibit osteogenic differentiation and mineralization of hDPCs through the β-catenin pathway, while simultaneously promoting their proliferation.^117^

Long non-coding RNAs (lncRNAs) are defined as transcripts exceeding 200 nucleotides in length, and some lncRNAs interact with PRC2 and direct it to specific sites in chromatin.^119^ In contrast, EZH2, a catalytic component of PRC2, have been observed to bind to RNA and, via EZH2-mediated trimethylation of H3K27, epigenetically silences KLF transcription, repress the expression of ALP, Osx, and OCN, and stimulate mineralization.^120^ A recent study has found that the mechanosensitive lncRNA SNHG8 exerts a regulatory effect on EZH2 in PDLSCs, thereby inhibiting osteogenic differentiation.^121^ Mechanical force has been shown to reduce EZH2 expression, accompanied by a delayed decrease in H3K27me3 expression and a significant decrease in H3K4me3 enrichment within the SNHG8 promoter region and 250 bp upstream of it. The results of ChIP-seq corroborated the mutual regulation of SNHG8 and EZH2 and demonstrated that the reduction in SNHG8 expression after mechanical force stretch was attributed to the diminution in the activation of its promoter. This indicated that the reduction in EZH2 expression was a consequence of decreased promoter activation. This indicates that the reduction of EZH2 is a pivotal epigenetic event in the mechanical response related to the maintenance of superenhancer stability and stem cell function. CARMN is an evolutionarily conserved lncRNA that is specific to smooth muscle cells and promotes the odontogenic differentiation of DPCs by impairing EZH2.^122^

#### KDM6A

UTX/KDM6A, UTY/KDM6C, and JMJD3/KDM6B are demethylases that remove methyl groups from H3K27me2 and H3K27me3, thereby activating the gene expression. Lysine-specific demethylase 6A (KDM6A), also known as UTX, is a member of the KDM6 family of H3K27 demethylases, with the gene predominantly located at Xp11.3. KDM6A contains a catalytic JmjC structural domain at the C terminus and six tetrapeptide repeat (TPR) structural domains at the N terminus. The catalytic JmjC structural domain enables KDM6A to demethylate H3K27me2/3. However, it has also been posited that the primary function of KDM6A is to recruit additional chromatin regulators.^123^ KDM6A is an important tumor suppressor^124^ that regulates a multitude of stem cell functions, including chondrogenesis, macrophage M2 differentiation, muscle differentiation, and neuronal differentiation. KDM6A has been shown to promote the osteogenic differentiation of PDLSCs by catalyzing H3K27me3 demethylation and enhancing the transcription of ALP, Runx2, and OPN.^125^ Inhibition of EZH2 may preserve the osteogenic potential of PDLSCs following KDM6A knockdown by regulating H3K27me3.^126^ The utilization of pharmacological agents that target the epigenetic regulation of the KDM6A gene has been demonstrated to effectively restore the osteogenic potential of cells. However, only a few studies have directly demonstrated alterations in KDM6A gene expression in patients with periodontitis.^52^ LncRNA HOTAIRM1 has been shown to upregulate KDM6A/B expression and suppress EZH2 in a HIF-1α-dependent manner, thereby reducing the distribution of H3K27me3 of ALP, M-CSF, Wnt-3a, Wnt-5a, Wnt-7a, β-catenin and others. This facilitates the transcription of these genes and promotes osteogenesis in hDFSCs.^127^

#### KDM6B

KDM6B, a histone demethylase with a Jmj-C structural domain, can convert H3K27me3 (repressive state) to H3K27me1 (activated state) within the central heterochromatin. Additionally, it has the potential to regulate gene transcription through demethylases in a demethylase-dependent or -independent manner. Human KDM6B, also known as JMJD3, contains a JmjC structural domain and a C-terminal fragment embedded in a GATA-like (GATAL) structural domain. JMJD3 counteracts the enzymatic activity of PRC2, thereby regulating the expression of specific genes. JMJD3 functions as a transcription factor that interacts with coactivators to regulate the transcription of target genes independent of its demethylase activity. Additionally, it facilitates transcriptional elongation and gene expression through its effect on RNA polymerase II (Pol II).^128^ KDM6B has been the subject of extensive studies in the context of immune diseases, cancer, and tumor development as well as in pluripotent stem cells and fate determination.^129,130^ The osteogenic differentiation of BM-MSCs is a process in which KDM6B is responsible for the removal of methyl groups from histones situated within the promoters of BMP2, BMP4, and HOXC6-1. This consequently regulates the expression of RUNX2.^100^ In the osteo-/odontogenic differentiation of DMSCs, KDM6B is recruited to the BMP2 promoter, resulting in the removal of the silencing genetic marker H3K27me3 and subsequent activation of transcription of the downstream odontogenic marker gene OSX/sp7, as well as the extracellular matrix genes BGLAP and OPN. The knockdown of KDM6B in DMSCs resulted in decreased ALP activity and mineralization.^131^ Postmenopausal patients with osteoporosis exhibit a parallel decline in the levels of these markers and mineralization, indicating a significant reduction in the population of bone cells and osteoblasts, as well as demineralization of both dense and cancellous bone. Although DPSCs primarily differentiate into dentin and BM-MSCs differentiate into bone, both dentin and bone formation share similar mineralized matrix components. The function of KDM6B is inhibited by alcohol, which results in impaired osteo/odontogenic differentiation of DPSCs and a reduction in the expression of several genes associated with mineralization, including BMP2, BMP4, OCN, and OPN. This indicates that excessive alcohol consumption may result in cellular damage associated with aberrant tooth development and osteoporosis.^132^ During the bell stage of human tooth embryo development, miR-93-5p was identified as a differentially expressed miRNA. It was observed to target KDM6B and regulate the H3K27me3 mark on the BMP2 promoter, thereby controlling odontogenic differentiation and dentin formation of DPSCs.^133^

#### Inflammatory condition

EZH2 and H3K27me3 expression was reduced in infected pulp tissues and cells. Inhibition of EZH2 suppressed the mRNA expression of IL-1β, IL-6, and IL-8 and proliferation in inflammation-stimulated hDPCs. Inhibition of EZH2 has been demonstrated to promote hDPCs mineralization through the epigenetic regulation of β-catenin expression and the activation of classical Wnt signaling pathways. LPS significantly upregulates EZH2 and H3K27me3 expression in hPDLSCs during the inflammatory process in periodontal tissues. Inhibition of EZH2 suppresses the LPS-induced upregulation of inflammatory factors (IL-6 and TNF-α) expression, cell proliferation and migration. In addition, the knockdown of EZH2 has been demonstrated to facilitate PDLSCs osteogenic differentiation by inhibiting the TLR4/MyD88/NF-κB signaling pathway.^118^ In the initial stages of periodontitis, KDM6B is recruited to the promoters of IL-6 and IL-12β, leading to H3K27me3 removal and the activation of target genes that regulate the inflammatory response.^134^ Insulin-like growth factors (IGFs) and their binding proteins (IGFBP5) have been linked to processes such as cell growth, bone repair, and remodeling. IGFBP5 has been identified as a downstream target gene of KDM6B, which forms a protein complex with the BCL6 co-repressor (BCOR). This complex may negatively regulate IGFBP5 transcription by promoting H3K27 methylation in the IGFBP5 promoter region. KDM6B can facilitate periodontal tissue regeneration by removing H3K27me3 from the IGFBP5 promoter region (Table 3).^135,136^

**Table 3:** Role of H3K27 histone methylation modification in Osteo-/Odontogenic differentiation of bone/dental-derived MSCs

Epigenetic modification factors		Epigenetic marker	Cell	Function	Reference
Normal	EZH2	H3K27me3↑	hDPCs	EZH2 inhibits the osteogenic differentiation and mineralization of hDPCs through the β-catenin pathway and promotes their proliferation	^117^
		H3K27me3↑	PDLSCs	LncRNA SNHG1 increases the level of H3K27me3 at the KLF2 promoter through the action of EZH2, thereby repressing the expression of ALP, Osx, and OCN	^120^
		H3K27me3↑	PDLSCs	LncRNAs SNHG8 and EZH2 reciprocally regulate each other to silence KLF transcription and inhibit osteogenic differentiation	^120,121^
		H3K27me3↑	hDPCs	LncRNA CARMN promotes odontogenic differentiation of hDPCs by inhibiting the activity of EZH2	^122^
	EZH2 KDM6A	H3K27me3	BM-MSCs	EZH2 and KDM6A co-regulate the expression of H3K27me3 in the promoter region of genes, thereby influencing the expression of Runx2, OPN, and OCN	^137^
	EZH2 KDM6A/B	H3K27me3	hDFSCs	LncRNA HOTAIRM1 promotes DFSCs-mediated bone regeneration by regulating the HIF1α/KDM6/EZH2/H3K27me3 axis	^127^
	KDM6B	H3K27me3↓	hDPCs	The transcription of downstream odontogenic marker genes, including OSX, BGLAP, and SPP1, is initiated	^100,131^
	KDM6B	H3K27me3↓	hDPSCs	The miR-93-5p targets KDM6B and removes the H3K27me3 marker on the BMP2 promoter, thereby regulating the odontogenic differentiation and dentin formation of DPSCs	^133^
Inflammatory conditions	EZH2	H3K27me3↑	PDLSCs	Knockdown of EZH2 promotes osteogenic differentiation by inhibiting the TLR4/MyD88/NF-κB signaling pathway	^118^
	KDM6B	H3K27me3↓	PDLSCs	KDM6B forms a protein complex with BCRO to inhibit IGFBP5 transcription. KDM6B removes H3K27me3 from the promoter region of the downstream gene IGFBP5 and mediates periodontal tissue regeneration	^136^

Trimethylation of H3K27 is a repressive epigenetic marker essential for the expression of genes involved in tooth development. The PRC2 core subunit, EZH2, catalyzes H3K27 trimethylation and maintains gene silencing, thereby inhibiting the osteogenic differentiation of DMSCs. The specific demethylase, KDM6A, enhances the osteogenic differentiation potential of DMSCs by removing the H3K27me3 tag at the Runx2 and Ocn transcription start sites. KDM6B is required for osteogenic differentiation, binds to BMP2 and HOX, and regulates the downstream transcription of OSX, BGLAP, and SPP1. Ezh2 and KDM6A are epigenetic switches that function in concert to regulate the level of H3K27me3 in the promoter region of genes, thereby affecting the expression of regulatory genes associated with adipogenesis and osteogenesis^137^ (Fig. 5c).

### Role of bivalent chromatin in osteo-/odontogenic differentiation of DMSCs

#### Bivalent chromatin

Bivalent chromatin is an epigenetic state that has been identified as a mechanism for lineage commitment and the regulation of developmental gene expression. It is characterized by the presence of H3K4me3 and H3K27me3. Chromatin immunoprecipitation demonstrated that H3K4me3 and H3K27me3 simultaneously occupy certain promoters in epithelial cells. Bivalent chromatin is indispensable for maintaining stem cell pluripotency. It maintains a balance between promoter accessibility and long-range junctions to regulate the expression of developmental genes, such as the regulation of VEGF-responsive angiogenesis^138^ and the timing of progenitor cell progression to mature neurons,^139^ affecting tooth organ development^65^. These modifications, designated as “bivalent structural domains,” serve to maintain a stable state for the genes in question, which subsequently determines whether they are activated or repressed during the process of differentiation. For example, under osteogenic induction conditions, H3K4me3 levels on the RUNX2 promoter in periodontal progenitor cells increased significantly 7 days after induction and then declined rapidly at 14 days. H3K4me3 and H3K27me3 markers on the OSX promoter exhibited a continued decline during osteogenic induction, and ChIP-seq results showed that the inhibitory histone markers H3K9me3 and H3K27me3 were higher in PDL progenitors. These data suggested that alterations in histone dynamics at the promoter level that repress key mineralizing transcription factors regulate mineralization in DMSCs.

#### H3K4 and H3K27

The presence of bivalent modifications characterized by H3K4me3 and H3K27me3 may exert a pivotal influence on tooth organogenesis by regulating cellular differentiation during tooth germ development. The expression of SET7 and EZH2, which function as methyltransferases, and KDM5B and JMJD3, which act as demethylases, was consistent with the observed expression of H3K4me3 and H3K27me3.^65^ Epigenetic regulators that modulate bivalent promoters include histone methyltransferases including Ezh2 and MLL1. Inhibition of Ezh2 has been observed to results in the redistribution of bivalent structural domains in transcriptional regulators related to the WNT and Hedgehog pathways in osteoblasts.^140^ Wnt5a, a significant member of the Wnt ligand family, is a lineage-specific gene that plays an important role in regulating the differentiation of DMSCs.^63^ It is markedly expressed in both mouse and human dental papillae and is associated with the regulation of odontogenic differentiation and dentin layer formation during the bell stage. WNT5A is associated with bivalent chromatin marks (H3K4me3/H3K27me3) in the stable state. Disassembly of H3K27me3 is a prerequisite for initiation of Wnt5a transcription. This process is subject to stringent regulation by the JMJD3 and MLL complexes, which ultimately determine cell fate commitment in hDPCs. Despite speculation about the involvement of Wnt signaling in the regulation of mineralization, there is a paucity of knowledge regarding the chromatin state of key promoters of mineralization genes, such as RUNX2 and OSX. Furthermore, the effect of these promoters on lineage commitment and matrix secretion in mineralized tissues remains unclear.^47^

#### H3K9 and H3K27

SATB2 is a transcription factor implicated in developmental regulation, chromatin remodeling, and transcriptional regulation. It plays a positive regulatory role in the differentiation of osteoblasts, bone formation, and regeneration of MSCs. Mutations in this gene often result in skeletal and dental hypoplasia.^141,142^ SATB2 promotes the osteogenic differentiation of PDLSCs, DPSCs, and stem cells isolated from human exfoliated deciduous teeth (SHEDs). JHDM1D/KDM7A is a histone demethylase that specifically removes the dimethylation marks of H3K9 and H3K27 from the promoters of target genes.^141,143^ SATB2 may regulate odontogenic differentiation of hDPSCs by downregulating DKK1 and activating the Wnt/β-catenin signaling pathway through the inhibition of JHDM1D expression. DKK1 is an important target of KDM6A/KDM7A,^125^ but the mechanism by which DKK1 regulates JHDM1D remains unclear.

#### H3K4 and H3K36

It has been demonstrated that inflammation and hypoxic niches exert an influence on MSC-mediated tissue regeneration. For instance, oxidative stress, characterized by abnormally elevated reactive oxygen species (ROS) levels, can induce mitochondrial dysfunction, leading to cell death. This, in turn, can diminish the osteogenic and odontogenic capacity of DPCs and intensify inflammatory states.^144,145^ Hypoxia inhibited the accumulation of superoxide in MSCs mitochondria, upregulated membrane potential, and internalized into damaged cells through extracellular vesicles, thus affecting metabolic status.^146^

KDM2A is a major member of the JmjC structural domain-containing histone demethylases, which are involved in the demethylation of H3K36 and H3K4, thereby regulating important biological processes, including chromatin remodeling and cell development. For instance, it is responsible for regulating the differentiation of enamel cells and odontoblasts.^147^ KDM2A has also been demonstrated to modify non-histone proteins, including β-catenin and NF-κB. This enables the regulation of their stability and transcriptional activity.^148^ KDM2A and BCOR have been shown to form a complex that inhibits osteogenesis. This is achieved by increasing histone H3K4/36 methylation of the epigenetic osteogenic protein (EREG) promoter. This in turn inhibits EREG transcription and subsequent OSX and DLX5 expression.^149^

SFRP2 is a classical extracellular inhibitor of Wnt signaling. It can also enhance the secretion of osteo-/odontogenic related factors such as IGFBP5, IGFBP4, MMP1, and cell homing related functional proteins CXCL5, CXCL12, CXCL6 in SCAP through paracrine effects. The demethylation process of SFRP2, catalysed by KDM2A, plays a regulatory role in the osteo-/odentigenic differentiation processes of SCAP.^150^ Under conditions of inflammation and hypoxia, SFRP2 inhibits NF-κB signal transduction by inhibiting Wnt/β-Catenin pathway and enhances osteo-/odontogenic differentiation of SCAP. In conditions of hypoxia, there is an increase in the expression of KDM2A and HIF-1, while BCOR expression remains unaltered. The transcription of SFRP2 is regulated by the demethylation of H3K36me2 and H3K4me3 on the SFRP2 promoter, which ultimately affects the osteo-/odontogenic differentiation function of SCAPs (Table 4).^151^

**Table 4:** Role of bivalent histone modifications in Osteo-/Odontogenic differentiation of bone/dental-derived MSCs

Epigenetic modification factors		Epigenetic marker	Cell	Function	Reference
Normal	MLL1 JMJD3	H3K4me3↑ H3K27me3↓	hDPCs	WNT5A is labeled by H3K4me3/H3K27me3 in the steady state, and the JMJD3 and MLL1 coactivator complexes mediate H3K27me3 resolution and initiation of transcription, ultimately regulating cell fate commitment in hDPCs	^63^
	KDM7A/JHDM1D	H3K9me2↓ H3K27me2↓	hDPSCs	SATB2 regulates DKK1 and KDM7A to modulate osteo-/odontogenic differentiation of hDPSCs through the Wnt/β-catenin signaling pathway	^141^
	NFIB-MLL1 KDM4B	H3K4me3↑ H3K9me3↓	C3H10T1/2	The NFIB-MLL1 complex mediated the deposition of H3K4me3 and resolution of H3K9me3 at the Dlx5 and Cebpa promoters and activated transcription of Runx2, Osx	^64^
	KDM2A	H3K36me2↓ H3K4me3↓	SCAP	KDM2A catalyzes the demethylation of H3K4me3 and H3K36me2 in the EGER promoter and inhibits osteogenesis through interaction with the BCOR complex	^149^
	KDM2A	H3K36me2↓ H3K4me3↓	SCAP	KDM2Ash promotes the transcription of the SFRP2 gene. SFRP2 enhances the osteo-/odontogenic differentiation potential of SCAP by activating OSX.	^150^
Inflammatory and hypoxic conditions	KDM2A	H3K36me2↓ H3K4me3↓	SCAP	KDM2A represses SFRP2 transcription and inhibits osteo-/odontogenic differentiation of SCAP. This repression is achieved by a reduction in the levels of H3K4me3 and H3K36me2 in the SFRP2 promoter region.	^151^

**Fig. 6:**
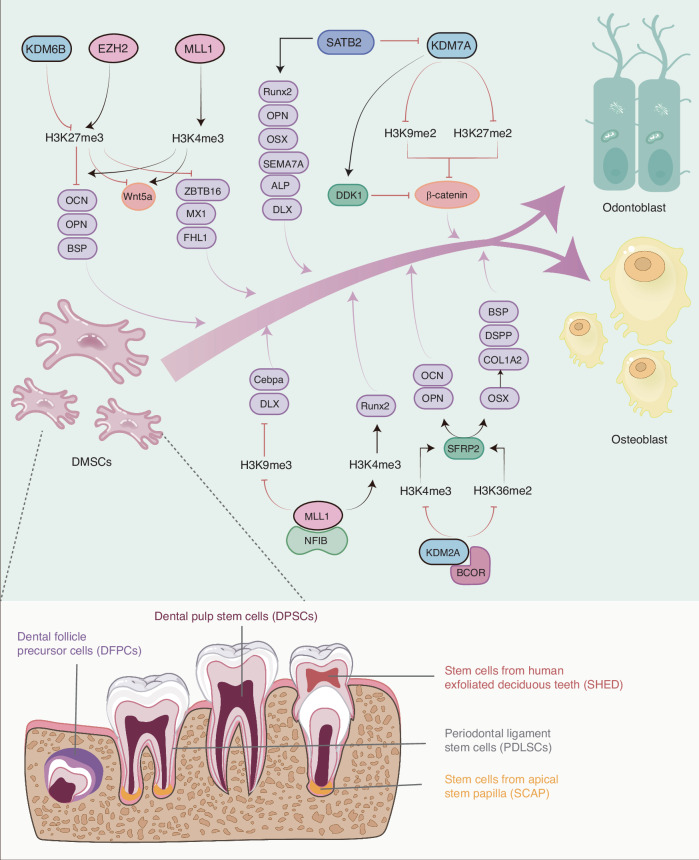
Role of bivalent histone modifications in the differentiation of bone/dental-derived mesenchymal stem cells

Despite the advent of massive parallel DNA sequencing techniques, numerous pivotal inquiries into bivalent promoters have persisted since the discovery of bivalence in pluripotent stem cells. To gain further insights into the unique patterns of H3K4me3 and H3K27me3 repair, it is essential to develop new methods that can accurately quantify subtle changes in multiple cell types. Further investigation may elucidate the potential links between bivalent chromatin and the osteo-/odontogenic differentiation of DMSCs, thereby advancing biomedical research on disease treatment and tissue regeneration (Fig. 6).

## Physiological and pathophysiological relevance of histone methylation regulation in DMSCs to health and disease in vivo

### The importance of histone methylation regulation of DMSCs in tooth development

During the development of tooth embryos, bivalent modifications are characterized by the presence of H3K4me3 and H3K27me3. The spatiotemporal expression of SET7, EZH2, KDM5B, and JMJD3 plays an instrumental role in this process.^65^ EZH2 has been shown to antagonize the activity of the Aridl protein, thereby regulating the process of formation and development of dental root forks in murine models through the action of Cdkn2a, which is a critical cell cycle inhibitor.^152^ ASH2L plays a significant role in the regulation of the expression of key developmental genes, including Shh and Trp63, by modulating H3K4me3 modification. Deficiencies in ASH2L have been observed to result in abnormal differentiation of tooth epithelium, which can ultimately manifest as defects in enamel development.^153^

### Osteoporosis

The gradual accumulation of epigenetic changes associated with the process of aging can lead to abnormal regulation of gene expression, metabolic instability, stem cell aging and/or failure, and tissue homeostasis imbalances.^154^ H3K4me3, H3K27me3, and H3K36me3 may have undergone ‘remodelling’ during ageing.^155^ Young exosomes secreted by SHED (SHED-Exos) regulate histone methylation and suppress NF-κB, thereby reversing the senescence of aged tendon stem/progenitor cells (AT-SC) and preserving their tenogenic capacity.^156^ α-ketoglutaric acid (α-KG), an intermediate derived from the tricarboxylic acid cycle, has demonstrated therapeutic potential in the treatment of age-related osteoporosis by reducing the accumulation of H3K9me3 and H3K27me3, upregulation of BMP signaling, and Nanog expression to restore MSCs function.^157^ Furthermore, serine synthesis-derived α-KG is imperative for the function of JMJD3, which catalyzes the removal of H3K27me3 at activated T nuclear factor, cytoplasmic 1 (Nfatc1) gene sites, consequently inducing NFATc1 expression and osteoclast maturation.^158^

The histone demethylase KDM7A has been shown to exerts a pivotal function in regulating bone homeostasis by modulating the differentiation of osteoblasts and osteoclasts. KDM7A has been observed to upregulate the expression of fibroblast activating protein alpha (FAP) and nuclear factor κB ligand receptor activator (RANKL) in BM-MSCs by removing H3K9me2 and H3K27me2 markers from FAP and RANKL promoters. Inhibition of KDM7A has been observed to result in increased FAP expression and inactivation of canonical Wnt signaling. Concurrently, this inhibition has been shown to promote osteoclast differentiation and bone resorption through enhanced RANKL expression.^159^

### Osteoarthritis

KDM6A has been shown to be involved in the process of chondrogenic differentiation of PDLSCs by demethylation of SOX9, Col2a1, and ACAN. The upregulation of KDM6A, or the application of EZH2 inhibitors, has been observed to have the potential to enhance the process of mesenchymal stem cell-mediated cartilage regeneration in cases of inflammatory tissue destruction, a condition that is exemplified by osteoarthritis.^126^

In osteoarthritis, a reduced methylation of lysine 79 on histone H3 (H3K79me) has been identified as a protective epigenetic mechanism. The study found that preservation of H3K79 methylation with KDM7A/B inhibitors, such as daminozide, was targeted to protect bone joints in intra-articular therapy in mice.^160^

KDM3A and G9A are a pair of antagonistic histone modification enzymes. KDM3A has been shown to attenuate the ubiquitination of SOX9 by demethylating lysine (K) 68 residues, thereby enhancing the stability of SOX9. In contrast, G9A has been shown to promote the ubiquitination and subsequent degradation of SOX9 by methylating K68 residues. The highly specific G9A inhibitor BIX-01294 has been shown to significantly induce the cartilage differentiation of DPSCs, providing a theoretical basis for enhancing the clinical application of DPSCs in cartilage tissue engineering therapy.^161^ Consequently, a combination strategy that targets multiple components of epigenetic mechanisms and utilizes a combination of synthetic interactions or immune blocking is expected to enhance the function of DMSCs.

## Potential therapeutic targets related to histone methylation regulation of DMSCs

### Vital pulp treatment

As previously stated, conventional dentistry is costly, intrusive, and based on a defunct mechanical understanding of dental diseases. In contrast, contemporary biological dentistry utilizes a cellular approach, focusing on the stimulation of cell activity and the promotion of regeneration.^162^ The development of epigenetic therapy drugs for pulp capping materials currently includes HDAC inhibitors, which have been shown to reduce inflammation and stimulate restorative dentin formation.^162,163^

However, there is still more that researchers need to learn about how changes to the building blocks of histone methylation affect the regeneration of pulp dentin complex. EZH2 can be used as a potential regulator of pulpitis and regeneration,^32,164^ and the development of inhibitors or small molecule agents to interfere with its function may be a viable potential therapeutic strategy.

### Orthodontic treatment

It has been demonstrated that the accumulation of advanced glycation end products (AGEs) in the periodontal tissue of type 2 diabetes patients contributes to enhanced bone fragility.^16^ This has been identified as a significant factor hindering orthodontic tooth movement and compromising the efficacy of orthodontic treatment. The targeting of the AGE/RAGE pathway or the enhancement of KDM6B function has been demonstrated to increase the antioxidant capacity of PDLSCs, inhibit cell senescence, and promote osteogenic differentiation. A combination of epigenetic or Wnt pathway modulators with RAGE blockers has the potential to enhance the efficacy of orthodontic treatment in patients with diabetes.^165^

### Maxillofacial bone defect

The treatment of maxillofacial bone defect resulting from periodontitis, trauma, and tumors is contingent upon the restoration of bone tissue.^166^ Following the degradation of bioactive microspheres, DP-Ak has been observed to release ions and activate sensory nerve cells, resulting in the secretion of calcitonin gene-related peptide (CGRP). The reduction of H3K27me3 levels, achieved by the inhibition of EZH2 and the enhancement of KDM6A, has been demonstrated to promote bone repair.^166^ The present study investigates the regulatory role of estrogen in DMSCs osteogenesis via the ERα/KDM6B/BMP2 axis in a rat cranioparietal defect model. This process is facilitated by the recruitment of KDM6B to the BMP2 and HOXC6 promoters, resulting in the removal of the H3K27me3 marker and subsequent activation of its transcription.^167^

## Summary and outlook

The methylation of histone lysine or arginine residues has been demonstrated to be of significant importance to gene regulation, as well as other physiological processes. Aberrant histone methylation, which can be precipitated by gene mutation, translocation, or overexpression, frequently results in the onset of developmental defects or diseases. MLL1/4, PRMT1/5, KDM5C, and KDM6B are essential for neurodevelopment, while EZH1/2, MLL4, KDM6B, and EED are implicated in cardiac development. LSD1, MLL1, EED, and G9a impact the hematopoietic system.^123^ Genetic mutations in H3K4 methyltransferases are linked to syndromes involving bone and facial deformities, intellectual impairment, and often reduced body size and microcephaly.^123^ Furthermore, it has been determined that mutations in other histone methylation regulators, such as EZH1 and NSD1, can be a causative factor for overgrowth syndrome. The utilisation of small molecule inhibitors of histone modifying enzymes, which correct abnormal methylation, has the potential to function as both novel therapeutic interventions for these diseases and as chemical probes for epigenetic studies.^168^ Nonetheless, the identification and advancement of small-molecule inhibitors of KMTs and KDMs remains in its nascent stages. Noteworthy endeavors and notable achievements from both academic and pharmaceutical sectors have only surfaced in recent years. Further experimentation is necessary to substantiate the pivotal function of histone methylation-related enzymes in disease processes and to establish a theoretical framework for enhancing the osteo-/odontogenic differentiation function of DMSCs or for the treatment of other diseases.

DMSCs play a pivotal role in tooth development and maintenance of oral tissue health. The capacity for differentiation and self-renewal is important in organizational engineering and regenerative medicine. The odontogenic differentiation of DMSCs is a crucial step in dentin formation. This process is orchestrated at the molecular level by a complex network of signaling pathways, transcription factors, and post-transcriptional and epigenetic regulatory mechanisms, which together facilitate the coordinated expression of a multitude of genes. The histone methylation status is a significant factor in stem cell differentiation. This paper reviews the role of epigenetic histone methylation modifications in tooth development and the potential mechanisms of histone methylation and demethylation enzymes in the regulation of the osteo-/odontogenic differentiation of DMSCs. Methylation of H3K4 and H3K36 is associated with the activation of transcription, whereas that of H3K9 and H3K27 is associated with the repression of transcription.

Osteogenesis comprises three distinct phases: proliferation, matrix maturation, and mineralization. These phases are regulated by transcription factors RUNX2 and OSX. RUNX2 and OSX are early markers of osteoblast/odontoblast differentiation, whereas OCN functions primarily during late osteogenesis. In MSCs, the expression of these genes is negatively regulated by inhibitory epigenetic markers including H3K4me1, H3K9me3, and H3K27me3. Simultaneous activation of the classical Wnt and BMP signaling pathways by RUNX2 and OSX is indispensable for formation and proliferation of the mature osteoblast phenotype. H3K4me2/3 marks were enriched in the promoter regions of osteo-/odontogenic genes of BM-MSCs and DMSCs. The inhibition of histone demethylases KDM1A and KDM5A expression has been demonstrated to facilitate the osteo-/odontogenic differentiation of DMSCs through the activation of Runx2, Osx, and DSPP gene transcription. However, under inflammatory conditions, the accumulation of H3K4me3 may also affect the expression of inflammatory genes. H3K9 methylation is primarily associated with the repression of gene transcription. KDM4B inhibits maxillofacial bone senescence, enhances the proliferation and migration of SCAP, and activates the expression of the osteogenic gene DLX5 by removing the H3K9me2/3 mark. The PRC2 core subunit EZH2 catalyzes the methylation of H3K27, thereby maintaining gene silencing. During the differentiation process of DMSCs, EZH2 and KDM6A/B function in concert to regulate the level of H3K27me3, thereby controlling the expression of osteo-/odontogenic-related genes, including BMP2, OSX, OCN, DSPP, and DMP1.

Bivalent histones may play a significant role in dental organ development and lineage-specific differentiation of MSCs by functioning as regulators of cell differentiation. Co-localization of H3K4me3 and H3K27me3 marks within the promoter region has been observed to alter the expression of certain genes. In pluripotent stem cells, the majority of H3K27me3 peaks were localized to H3K4me3-tagged promoters, indicating that genes with bivalent structural domains are the prevailing phenomenon rather than an exception. This phenomenon also applies to osteogenic differentiation of DMSCs. The inhibitory epigenetic markers H3K9me3 and H3K27me3 exhibit a pattern of bivalent modifications in dental mesenchymal progenitor cells and are predominantly located on OSX during odontogenic differentiation. It has been hypothesized that the histone methylases EZH2, MLL1, and MLL4, in conjunction with the histone demethylases KDM6A/B and KDM7A, may coordinately control the levels of H3K4me3, H3K9me3, and H3K27me3 in specific gene promoters, thereby controlling downstream gene expression. Although most studies have been conducted in vitro, further in vivo studies and animal disease models are necessary to evaluate their potential applications in disease treatment and regenerative medicine. In addition to histone modifications, a number of other epigenetic modifications, including those of RNA and molecular chaperones, contribute to a range of biological processes, including embryonic development, cell differentiation, and maintenance of pluripotency. In conclusion, dynamic modulation of histone methylation plays a significant and multifaceted role in the regulation of chromatin state. Modulating histone functions to control the differentiation of DMSCs is a promising approach for achieving tooth regeneration.

## Supplementary information

Plagiarism_Check-2
