# Supplementary material for: Role and mechanisms of histone methylation in osteogenic/odontogenic differentiation of dental mesenchymal stem cells
Source: Int J Oral Sci. 2025 Mar 26;17:24. doi: 10.1038/s41368-025-00353-z (PMC11937254; doi:10.1038/s41368-025-00353-z)
Supplement: Supplementary file 1 — Plagiarism_Check-2 [file 41368_2025_353_MOESM1_ESM.pdf]

115678

Submission date: 01-Jan-2025 12:28AM (UTC-0800)
Submission ID: 2559143670
File name: .docx (42.64K)
Word count: 1910
Character count: 11834

Mesenchymal stem cells (MSCs) are a population of stem cells that function as self-renewing and differentiating progenitor cells. The International Society for Cell & Gene Therapy(ISCT) Mesenchymal Stromal Cell committee defines them as readily adherent, expressing CD105, CD73 and CD90 (but not CD11b, CD14, CD19, CD34, CD45, CD79a, HLA-DR), and capable of differentiating in vitro into osteoblasts, chondroblasts, and adipocyte lineages.[3,4]

Epigenetic modifications can be divided into three main categories: DNA methylation, histone modifications, and chromatin remodelling. DNA methylation is primarily associated with gene regulation and can maintain chromosome integrity. Histone modification plays an important role in a variety of biological processes, including transcriptional regulation, DNA repair, DNA replication, alternative splicing, and chromosome aggregation.[164]

Wnt signaling has been demonstrated to regulate the function of Runx1 and Runx2.26 The canonical Wnt/β-catenin signalling pathway has been shown to regulate osteoblast differentiation. This pathway involves the binding of Wnt to the FZD receptor, which results in the accumulation of β-catenin. The subsequent movement of β-catenin to the nucleus leads to the transcription of target genes.26 The noncanonical Wnt signaling ligand, WNT5A, has been identified as a key lineage-specific gene in the regulation of differentiation of dental mesenchymal cells. FGFs are a family of secreted peptides controlling a series of secreted peptides in intrachondral and intramembranous ossification.

Upon activation of Smads (Smad1/5/8), BMP2 initiates runx2 gene transcription through its distal P1 promoter and proximal P1 promoter. Runx2 is also phosphorylated by the non-canonical BMP signaling pathway (TAK1-MEK-p38 or ERK), which promotes its association with the co-activator CREB binding protein (CBP).[163] In addition, BMP also promotes Runx2 acetylation via p300 for Runx2 stabilization.[163] The MLL1/2 complex contains the tumor suppressor Menin, which facilitates the recruitment of the MLL1/2 complex to HOX and other sites. The MLL3/4 complex contains NCOA6, PTIP, and PA-1, which contributes to the targeting of the MLL3/4 complex to a distinct set of genes. The MLL3/4 complex also comprises the H3K27 demethylase UTX.[55]

The phytohormone Ferutinin has been shown topromote the expression of osteocalcin and collagen 1A1 mRNA and protein levels by activating H3K9 acetylation and H3K4 trimethylation markers in the promoter regions of WNT3A and DVL3 and promote the osteogenic differentiation of DPSCs.

During the process of inflammation, various inflammatory factors (e.g., TNF-a, IL-1, IL-6) have been observed to affect the epigenetic characteristics of cells, thereby influencing gene expression.[77] Moreover, the inflammatory microenvironment has been demonstrated to impact stem cell differentiation and function.

In patients with chronic periodontitis, bacteria can alter histone modifications in periodontal tissue and activate a series of inflammatory and bone metabolism-related signaling pathways[78,79]. Lipopolysaccharide (LPS) has been identified as a pivotal periodontal pathogenic factor, capable of activating NF-κB signaling and promoting the release of inflammatory cytokines such as IL-6 and IL-8. Research has demonstrated that rat dental capsule stem cells possess the capacity to modulate the expression of ERK1/2 and NF-κB signaling pathway via the paracrine pathway. This modulation involves the repression of IL-1β, IL-6, and TNF-α gene expression, while concurrently promoting the expression of IL-4 and TGF-β. This regulatory mechanism contributes to the preservation of dental pulp regeneration in rats afflicted with inflammation.[80]

It has been demonstrated that inflammation and hypoxic niches exert an influence on MSC-

mediated tissue regeneration. For instance, oxidative stress, characterized by abnormally elevated ROS (reactive oxygen species) levels, can induce mitochondrial dysfunction, leading to cell death. This, in turn, can diminish the osteogenic and odontogenic capacity of DPCs and intensify inflammatory states.[142,143] Hypoxia inhibited the accumulation of superoxide in MSCs mitochondria, upregulated membrane potential, and internalized into damaged cells through extracellular vesicles, thus affecting metabolic status.[144]

SFRP2 is a classical extracellular inhibitor of Wnt signaling. It can also enhance the secretion of osteo-/odontogenic related factors such as IGFBP5, IGFBP4, MMP1, and cell homing related functional proteins CXCL5, CXCL12, CXCL6 in SCAPs through paracrine effects. The demethylation process of SFRP2, catalysed by KDM2A, plays a regulatory role in the differentiation processes of osteo-/odentigenic SCAPs.[148] Under conditions of inflammation and hypoxia, SFRP2 inhibits NF-κB signal transduction by inhibiting Wnt/β-Catenin pathway and enhances osteo-/odontogenic differentiation of SCAPs. In conditions of hypoxia, there is an increase in the expression of KDM2A and HIF-1, while BCOR expression remains unaltered. The transcription of SFRP2 is regulated by the demethylation of H3K36me2 and H3K4me3 on the SFRP2 promoter, which ultimately affects the osteo-/odontogenic differentiation function of SCAPs.

3 Physiological and pathophysiological relevance of histone methylation regulation in DMSCs to health and disease in vivo.

3.1 The importance of histone methylation regulation of DMSC in tooth development

During the development of tooth embryos, bivalent modifications are characterized by the presence of H3K4me3 and H3K27me3. The spatiotemporal expression of SET7, EZH2, KDM5B, and JMJD3 plays an instrumental role in this process.[63] EZH2 has been shown to antagonize the activity of the Aridl protein, thereby regulating the process of formation and development of dental root forks in murine models through the action of Cdkn2a, which is a critical cell cycle inhibitor.[150] ASH2L plays a pivotal role in the regulation of the expression of key developmental genes, including Shh and Trp63, by modulating H3K4me3 modification. Deficiencies in ASH2L have been observed to result in abnormal differentiation of tooth epithelium, which can ultimately manifest as defects in enamel development.[151]

3.2 Osteoporosis

The gradual accumulation of epigenetic changes associated with the process of aging can lead to abnormal regulation of gene expression, metabolic instability, stem cell aging and/or failure, and tissue homeostasis imbalances.[152] H3K4me3, H3K27me3 and H3K36me3 may have undergone 'remodelling' during ageing.[153] Young exosomes secreted by SHED (SHED-Exos) regulate histone methylation and inhibit NF-κB to reverse senescence of aged tendon stem/progenitor cells (AT-SC) and maintain their tenogenic capacity.[154] α-ketoglutaric acid (α-KG), an intermediate derived from the tricarboxylic acid cycle, has demonstrated therapeutic potential in the treatment of age-related osteoporosis by reducing the accumulation of H3K9me3 and H3K27me3, upregulation of BMP signaling, and Nanog expression to restore MSC function.[155] Furthermore, serine synthesis-derived α-KG is imperative for the function of JMJD3, which catalyzes the removal of H3K27me3 at activated T nuclear factor, cytoplasmic 1 (Nfatc1) gene sites, consequently inducing NFATc1 expression and, consequently, osteoclast maturation.[156]

The histone demethylase KDM7A has been shown to play a critical role in regulating bone

homeostasis by modulating the differentiation of osteoblasts and osteoclasts. KDM7A has been observed to up-regulate the expression of fibroblast activating protein alpha (FAP) and nuclear factor κB ligand receptor activator (RANKL) in bone marrow mesenchymal stem cells by removing H3K9me2 and H3K27me2 markers from FAP and RANKL promoters. Inhibition of KDM7A has been observed to result in increased FAP expression and inactivation of canonical Wnt signaling. Concurrently, this inhibition has been shown to promote osteoclast differentiation and bone resorption through enhanced RANKL expression.[157]

3.3 Osteoarthritis

KDM6A has been shown to be involved in the process of chondrogenic differentiation of PDLSCs by demethylation of SOX9, Col2a1, and ACAN. The upregulation of KDM6A, or the application of EZH2 inhibitors, has been observed to have the potential to enhance the process of mesenchymal stem cell-mediated cartilage regeneration in cases of inflammatory tissue destruction, a condition that is exemplified by osteoarthritis. [124]

In osteoarthritis, a reduced methylation of lysine 79 on histone H3 (H3K79me) has been identified as a protective epigenetic mechanism. The study found that preservation of H3K79 methylation with KDM7A/B inhibitors, such as daminozide, was targeted to protect bone joints in intra-articular therapy in mice.[158]

KDM3A and G9A are a pair of antagonistic histone modification enzymes. KDM3A has been shown to attenuate the ubiquitination of SOX9 by demethylating lysine (K) 68 residues, thereby enhancing the stability of SOX9. In contrast, G9A has been shown to promote the ubiquitination and subsequent degradation of SOX9 by methylating K68 residues. The highly specific G9A inhibitor BIX-01294 has been shown to significantly induce the cartilage differentiation of DPSCs, providing a theoretical basis for enhancing the clinical application of DPSCs in cartilage tissue engineering therapy.[159]Consequently, a combination strategy that targets multiple components of epigenetic mechanisms and utilizes a combination of synthetic interactions or immune blocking is expected to enhance the function of DMSCs.

4 Potential therapeutic targets related to histone methylation regulation of DMSCs

4.1 Vital Pulp Treatment

As previously stated, conventional dentistry is costly, intrusive, and based on a defunct mechanical understanding of dental diseases. In contrast, contemporary biological dentistry utilizes a cellular approach, focusing on the stimulation of cell activity and the promotion of regeneration. [160] The development of epigenetic therapy drugs for pulp capping materials currently includes HDAC inhibitors, which have been shown to reduce inflammation and stimulate restorative dentin formation.[160,161]

However, there is still more that researchers need to learn about how changes to the building blocks of histone methylation affect the regeneration of pulp dentin complex. EZH2 can be used as a potential regulator of pulpitis and regeneration[162,163], and the development of inhibitors or small molecule agents to interfere with its function may be a viable potential therapeutic strategy.

4.2 Orthodontic treatment

Recent studies have demonstrated that the accumulation of advanced glycation end products (AGEs) in the periodontal tissue of type 2 diabetes patients contributes to enhanced bone fragility.[164] This has been identified as a significant factor hindering orthodontic tooth movement and compromising the efficacy of orthodontic treatment. The targeting of the AGE/RAGE pathway or the enhancement of KDM6B function has been demonstrated to increase the antioxidant capacity of PDLSCs, inhibit cell senescence, and promote osteogenic differentiation. A combination of epigenetic or Wnt pathway modulators with RAGE blockers has the potential to enhance the efficacy of orthodontic treatment in patients with diabetes. [165]

4.3 Maxillofacial Bone Defect

The treatment of maxillofacial bone defects resulting from periodontitis, trauma, and tumors is contingent upon the restoration of bone tissue.[166] Following the degradation of bioactive microspheres, DP-Ak has been observed to release ions and activate sensory nerve cells, resulting in the secretion of calcitonin gene-related peptide (CGRP). The reduction of H3K27me3 levels, achieved by the inhibition of EZH2 and the enhancement of KDM6A, has been demonstrated to promote bone repair.[166] The present study investigates the regulatory role of estrogen in DMSC osteogenesis via the ERα/KDM6B/BMP2 axis in a rat cranioparietal defect model. This process is facilitated by the recruitment of KDM6B to the BMP2 and HOXC6 promoters, resulting in the removal of the H3K27me3 marker and subsequent activation of its transcription.[167]

5 Summary and outlook

Methylation of histone lysine or arginine residues plays an important role in gene regulation and other physiological processes. Aberrant histone methylation, precipitated by gene mutation, translocation, or overexpression, frequently results in the onset of developmental defects or diseases. MLL1/4, PRMT1/5, KDM5C, and KDM6B are essential for neurodevelopment, while EZH1/2, MLL4, KDM6B, and EED are implicated in cardiac development. LSD1, MLL1, EED, and G9a impact the hematopoietic system.[121] Genetic mutations in H3K4 methyltransferases are linked to syndromes involving bone and facial deformities, intellectual impairment, and often reduced body size and microcephaly.[121] Mutations in other histone methylation regulators, like EZH1 and NSD1, can cause overgrowth syndrome. Small molecule inhibitors of histone modifying enzymes that correct abnormal methylation could be used as new therapies for these diseases or as chemical probes for epigenetic studies.[168] Nonetheless, the identification and advancement of small-molecule inhibitors of HMTs and KDMs remains in its nascent stages. Noteworthy endeavors and notable achievements from both academic and pharmaceutical sectors have only surfaced in recent years. Further experimentation is necessary to substantiate the pivotal function of histone methylation-related enzymes in disease processes and to establish a theoretical framework for enhancing the osteo-/odontogenic differentiation function of DMSCs or for the treatment of other diseases.

115678

ORIGINALITY REPORT

19%
SIMILARITY INDEX

PRIMARY SOURCES

1	www.ncbi.nlm.nih.gov<br>Internet Source	4%
2	E. Smith, C. Lin, A. Shilatifard. "The super elongation complex (SEC) and MLL in development and disease", Genes & Development, 2011<br>Publication	2%
3	www.frontiersin.org<br>Internet Source	1%
4	www.researchgate.net<br>Internet Source	1%
5	Submitted to University of Witwatersrand<br>Student Paper	1%
6	Shanshan Jin, Yu Wang, Xiaolan Wu, Zixin Li, Lisha Zhu, Yuting Niu, Yongsheng Zhou, Yan Liu. "Young Exosome Bio-Nanoparticles Restore Aging-Impaired Tendon Stem/Progenitor Cell Function and Reparative Capacity", Advanced Materials, 2023<br>Publication	1%

7 Ye Liu, Wei Xiong, Junyi Li, Huixian Feng, Shuili Jing, Yonghao Liu, Heng Zhou, Duan Li, Dehao Fu, Chun Xu, Yan He, Qingsong Ye. "Application of dental pulp stem cells for bone regeneration", Frontiers in Medicine, 2024 — 1 %
Publication

8 Yuexin Xu, Jing Ma, Guohua Xu, Duan Ma. "Recent advances in the epigenetics of bone metabolism", Journal of Bone and Mineral Metabolism, 2021 — 1 %
Publication

9 de Rooy, D. P. C., N. G. Yeremenko, A. G. Wilson, R. Knevel, E. Lindqvist, T. Saxne, A. Krabben, M. K. Leijsma, N. A. Daha, S. Tsonaka, A. Zhernakova, J. J. Houwing-Duistermaat, T. W. J. Huizinga, R. E. M. Toes, D. L. P. Baeten, E. Brouwer, and A. H. M. van der Helm-van Mil. "Genetic studies on components of the Wnt signalling pathway and the severity of joint destruction in rheumatoid arthritis", Annals of the Rheumatic Diseases, 2012. — 1 %
Publication

10 Submitted to University of Birmingham — 1 %
Student Paper

11 Zhenqing Liu, Hye-Lim Lee, Jin Sook Suh, Peng Deng et al. "The ERα/KDM6B regulatory axis modulates osteogenic differentiation in — 1 %

human mesenchymal stem cells", Bone Research, 2022
Publication

12	Submitted to Roehampton University<br>Student Paper	1 %
13	res.mdpi.com<br>Internet Source	1 %
14	www.sciencegate.app<br>Internet Source	1 %
15	Submitted to Istanbul Medipol Ãniversitesi<br>Student Paper	<1 %
16	Chengyuan Xie, Lu Huang. "How to drive sustainable economic development: The role of fintech, natural resources, and social vulnerability", Resources Policy, 2024<br>Publication	<1 %
17	Daniela N. Rolph, Moonmoon Deb, Suman Kanji, Carl J. Greene et al. "Ferutinin directs dental pulp-derived stem cells towards the osteogenic lineage by epigenetically regulating canonical Wnt signaling", Biochimica et Biophysica Acta (BBA) - Molecular Basis of Disease, 2018<br>Publication	<1 %
18	rcastoragev2.blob.core.windows.net<br>Internet Source	<1 %

19	www.tdx.cat<br>Internet Source	<1%
20	Ashwini Jambhekar, Abhinav Dhall, Yang Shi. "Roles and regulation of histone methylation in animal development", Nature Reviews Molecular Cell Biology, 2019<br>Publication	<1%

Exclude quotes	Off	Exclude matches	Off
Exclude bibliography	Off		
